# On the Applicability of Brain Reading for Predictive Human-Machine Interfaces in Robotics

**DOI:** 10.1371/journal.pone.0081732

**Published:** 2013-12-16

**Authors:** Elsa Andrea Kirchner, Su Kyoung Kim, Sirko Straube, Anett Seeland, Hendrik Wöhrle, Mario Michael Krell, Marc Tabie, Manfred Fahle

**Affiliations:** 1 Robotics Lab, University of Bremen, Bremen, Germany; 2 Robotics Innovation Center (RIC), German Research Center for Artificial Intelligence (DFKI), Bremen, Germany; 3 Brain Research Institute IV, University of Bremen, Bremen, Germany; 4 The Henry Wellcome Laboratories for Vision Sciences, City University, London, United Kingdom; University of British Columbia, Canada

## Abstract

The ability of today's robots to autonomously support humans in their daily activities is still limited. To improve this, *predictive* human-machine interfaces (HMIs) can be applied to better support future interaction between human and machine. To infer upcoming context-based behavior relevant brain states of the human have to be detected. This is achieved by *brain reading* (BR), a passive approach for single trial EEG analysis that makes use of supervised machine learning (ML) methods. In this work we propose that BR is able to detect concrete states of the interacting human. To support this, we show that BR detects patterns in the electroencephalogram (EEG) that can be related to event-related activity in the EEG like the P300, which are indicators of concrete states or brain processes like target recognition processes. Further, we improve the robustness and applicability of BR in application-oriented scenarios by identifying and combining most relevant training data for single trial classification and by applying classifier transfer. We show that training and testing, i.e., application of the classifier, can be carried out on different classes, if the samples of both classes miss a relevant pattern. Classifier transfer is important for the usage of BR in application scenarios, where only small amounts of training examples are available. Finally, we demonstrate a dual BR application in an experimental setup that requires similar behavior as performed during the teleoperation of a robotic arm. Here, target recognition processes and movement preparation processes are detected simultaneously. In summary, our findings contribute to the development of robust and stable predictive HMIs that enable the simultaneous support of different interaction behaviors.

## Introduction

During the last decades different approaches were developed to support humans in their daily life and working environment or to restore sensory and motor functions with the help of intelligent and autonomous robotic systems that behave situational and support humans according to the context [1]–[5]. However, autonomous systems do not yet come close to the cognitive capabilities of humans regarding their ability to react mostly correctly and appropriately to a new situation. Therefore, the application of robotic systems is to some degree restricted to certain situations and environments.

Some approaches solve restrictions of autonomous robotic behavior by using human-machine interfaces (HMIs). HMIs for example *explicitly* send commands to robots when autonomous behavior cannot handle a given situation as shown by, e.g., Kaupp et al. [2] for teleoperation. However, to explicitly send a control command requires enhanced cognitive demands by the interacting human. Between humans, *implicit* information is transferred beside explicit information during interaction that can be used by the interacting persons to *infer* on the general state of each other, like the emotional state, involvement in the interaction or communication or the mental load. This implicit information serves to adapt behavior to interact better, e.g., more efficiently. Thus, a promising approach for improving the behavior of autonomous artificial systems is to adapt them with respect to the state of the interacting human. Such adaptation of technical systems is in a more general sense also known as biocybernetic adaptation [6]. It is usually used to, e.g., change the functionality of a system regarding fatigue or frustration levels of a user and can enable better control over complex systems [7]. For this aim (psycho-)physiological data from the user like galvanic skin response, blood pressure, gesture, eye gaze, mimic, prosody, brain activity or combinations of those are applied [5], [6], [8], [9].

### Establishing and Supporting Interaction by Brain-Computer Interfaces

The human's EEG has been used since some decades to develop brain-computer interfaces (BCIs) with the goal to (re-)establish explicit interaction and communication [10]–[15]. For this purpose, active and reactive BCIs enable the user to control a computer or machine via the nervous system and can replace classical HMIs for the explicit control of devices like keyboard, mouse or joystick. They were mainly developed to open up new ways of communication for disabled persons [10], [11], [16], for example, to control a speller by imaging hand movements [17]. Recently, active and reactive BCIs are also used by healthy people [18], e.g., in BCI controlled computer games [19], [20]. Active and reactive BCIs have some main drawbacks in their application: The user has to concentrate on the task of controlling the device via his brain activity, hence the application of such BCIs typically requires a high amount of cognitive resources from the user. However, training can improve, even automate the control of such BCI and thus reduce the effort. Further, due to the direct link between brain and machine, misclassifications of the brain signals always have an impact on the application and can lead to faulty behavior [21] or inaccuracies. There are, however, promising approaches that attempt to automatically correct misclassifications in active BCIs. For example, [22], [23] have shown that misclassifications of brain activity can be compensated by autonomous interpretation of the situation by the cooperating robotic system.

To extend the usage of EEG activity for physiological computing [6] *passive* or *implicit* BCIs were developed [24], [25]. They have their roots in several approaches in the past that focus on user-state detection [25], [26]. For example, in [25] the detection of error potentials is used to correct errors that happen during a rotation task which is performed by the application of an active human-computer interface (HCI) that is manipulated in a way that execution errors are introduced randomly. Since users of passive or implicit BCIs do not actively influence their brain activity, i.e., do not explicitly control a device by brain activity and do not actively produce brain activity, they seem to be an appropriate tool to improve human-machine interaction by implicitly gained information about the humans brain state. It was further proposed that passive BCIs can be integrated into more complex and natural control systems, like emergency braking assistance in cars to improve their functionality. Haufe et al. 2011 [27] discuss that an emergency braking assistance system could be modified by predicting upcoming braking behavior based on EEG analysis. The given examples furthermore show that compared to active or reactive BCIs, passive BCIs seem to be even more easily applicable in hybrid HMI or BCI approaches [28], [29], where at least two different kinds of BCIs or a HMI and a BCI as in [25] are combined.

### Embedded Brain Reading in Robotic Applications

Our approach to improve interaction in robotic application scenarios was to implement *embedded brain reading* (eBR) [30]. It allows to integrate implicitly gained information about the human from his brain's activity into the control of HMIs to automatically adapt them for a better support of future interaction behavior. Since such HMIs are adapted by eBR with respect to inferred *upcoming* interaction behavior we call the resulting HMIs *predictive* HMIs. Since we make use of implicit information, our approach is similar to the approach of passive BCI, however we focus on applications in which upcoming interaction behavior can be supported instead of, e.g., correcting former false behavior. In eBR the detection of specific brain patterns by means of machine learning (ML) methods and the process of relating them to specific states of the user, e.g., his intentional state, is called *brain reading* (BR). BR was introduced as a method to gain information about hidden processes and states of the brain, i.e., the function of the mind [31]. BR can even be applied to detect different conscious states of the human, i.e., in his conscious perception [32]. However, more functional questions like the decoding of visual, auditory, perceptual or cognitive patterns are addressed as well [33]–[37]. For our purpose, we define BR as the passive decoding of brain activity, i.e., detection of certain brain patterns that are related to specific functional, cognitive or intentional (but not necessarily conscious) processes, which are evoked by internal or external events during human-machine interaction. BR takes place unnoticed by the user and requires no extra attentional or cognitive resources of the user it is applied to.

**Figure 1:**
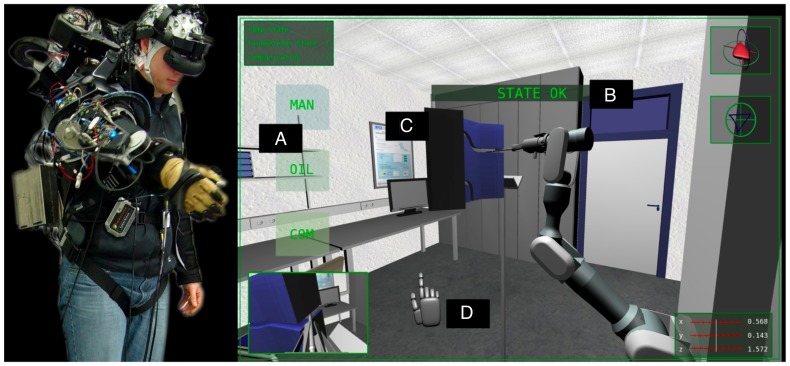
Experimental setup for the teleoperation scenario – a holistic feedback control of semi-autonomous robots. In the teleoperation scenario an operator is wearing an exoskeleton and, with the support of a virtual scenario, is tele-manipulating a robotic arm. A: three kinds of virtual response cubes (different responses are required for different types of warnings); B: different kinds of stimuli: unimportant stimulus (STATE OK – no response required), warning (first target – response required), repeated and enhanced warning (second target – response required), third warning (response is critical, e.g., exoskeleton control is disabled); C: labyrinth that the robot has to be moved through; D: virtual hand.

The application of eBR to adapt HMIs and the tasks of BR can be explained on the example of a robotic telecontrol scenario (see Fig. 1), where two HMIs are implemented for human-machine interaction. During teleoperation the operator has to understand information about the general situation or possible hazards, e.g., a person entering the operating area of the robot, a malfunction of the exoskeleton or robot, or requests for communication from outside, such as a second task. It is known that under such conditions of high workload attention to a second task can be impaired [7], [38]. This impairment can lead to failure in one of the tasks, most likely the subjectively less important one. Since manipulation of the exoskeleton requires a very high amount of the user's cognitive resources, it is very likely that he misses important information. It is well known that the event-related potential (ERP) P300 is evoked whenever the brain detects information that appears infrequently in the user's subjective perception. Several sub-components of the positive P300 are known, i.e., novelty P3, P3a and P3b [39]–[41]. The P3b component is evoked by infrequent task relevant stimuli and is therefore not only an indicator for attentional, but also for early cognitive processes, i.e., when target evaluation and recognition takes place [41]–[44]. The amplitude of the P300 does not only depend on the subjective impression of the frequency of occurrence of stimuli but also on the importance of a presented stimulus and whether a subject devotes high amounts of effort to the task [38]. A reduction of the amplitude of P300 can be found in case of ambiguous stimuli for which relevance and importance might not be clear. In case that a subject misses an important stimulus it is expected that no P300 is expressed [45]. Since in the teleoperation scenario a dual-task (controlling the robot by the exoskeleton and responding to important information) is performed, it can further be assumed that besides brain activity related to target recognition processes also other partly overlapping ERP components related to the retrieval of intended action from long-term memory, post-retrieval monitoring, and task coordination processes will be evoked by target stimuli and accompanied by further EPRs like the prospective positivity [46]–[48].

**Figure 2:**
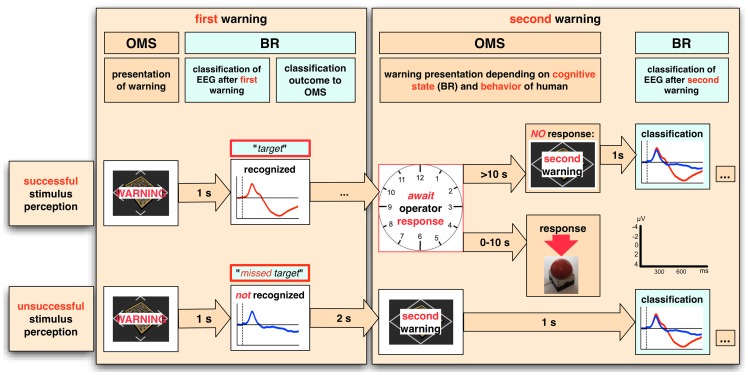
Adaptation of an operator monitoring system by BR. The currently implemented message scheduling procedure which is controlled by the operator monitoring system (OMS) is shown. The OMS considers the cognitive state that is detected by BR and allows to infer the behavior of the human. The general procedure is described in the following: After a warning the operator's EEG is analyzed by BR. Detection of successes versus no success in the recognition of important information by BR allows to infer future behavior (response or no response) by eBR. As a consequence, the behavior of the OMS is adapted, i.e., the tolerated response time is extended or a second warning is presented right away by the OMS. In case the operator does not respond to the second warning, a third warning follows. Approximate time required for predictions made by BR and predefined response times are given in the arrows.

Hence, in the teleoperation scenario we used ERP activity, i.e., positive parietal ERP activity, mainly the P300. Instead of having a second person to assist the operator we adapted the implemented operator monitoring system (OMS) by eBR to better assist the operator under both conditions, i.e., if she/he recognized an important warning or did not recognize it. The task of BR was to detect different brain patterns, i.e., patterns that were evoked by the recognition of important stimuli (that contain a P300) and patterns that were evoked by important stimuli that were not recognized, i.e., missed (containing *no* P300). This information was then used to infer whether or not the operator would respond and to adapt the repetition time for warnings appropriately by eBR. For example, if eBR infers that the operator will respond (in case BR detected brain patterns related to the recognition of important stimuli) the tolerated response time is extended. On the other hand, if eBR infers that the operator will *not* respond (in case BR did *not* detect brain patterns related to the recognition of important stimuli) the allowed response time is reduced or the important information is repeated immediately (see Video S1 and Fig. 2). Experiments conducted so far support our approach [49]. Subjects reported that an adapted OMS can reduce stress by avoiding to force fast responses and emphasizes important information by repeating them at a higher frequency in case the subject was distracted.

**Figure 3:**
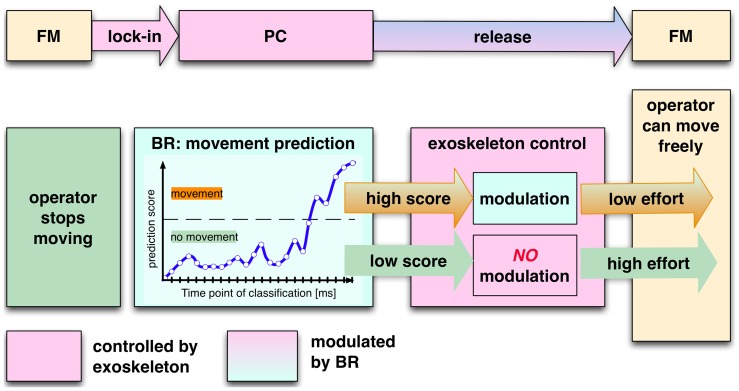
Adaptation of the exoskeleton's control by BR. It is shown how BR adapts the exoskeletons control. The exoskeleton is supporting the user while moving (free mode: FM). In case the user stops moving, the exoskeleton will lock in to support the arm at a chosen position (position control mode: PC). For release the user has to press against sensors that are integrated into the exoskeleton. To ease the release BR detects movement intention. The movement prediction score is then used to modulate the exoskeleton's control by eBR: the higher the prediction score (i.e., the more certain the classifier is) the stronger is the adaptation of the exoskeleton's control and the lower is the effort for the user to transfer the exoskeleton from PC to FM mode. Pressure against the sensors is always required for release, which is minimizing the risk of false lock out in case of possible false detection of movement intention by BR. Adapted from [52].

A central part of the teleoperation scenario (see Fig. 1) is an exoskeleton developed by our group [50] to intuitively control different robotic arms or legs [51], [52]. The exoskeleton used for teleoperation serves both as a control device for a semi-autonomous robot as well as an interface for the control of a virtual scenario (for visualization of the scenario see Video S2). For control reasons the switch between two operating modes of the exoskeleton: (i) a position control mode (PC) where the exoskeleton supports the user, i.e., by allowing him to rest and (ii) a free run mode (FM) where the operator can move freely and control the virtual scenario (see Fig. 3 adopted from [52]) is very interesting for an adaptation by eBR and could be shown to be applied successfully [53]. During rest the applied control mechanism of the exoskeleton cannot make predictions about upcoming behavior as it is possible during interaction [54]. To improve interaction it is relevant to know whether the operator wants to move again.

Movement intention can be predicted from the user's EEG. Kornhuber and Deecke [55] showed that a complex of ERPs precedes intended movements. Most prominent are the Bereitschaftspotential (BP) or Readiness Potential (RP) and the Lateralized Readiness Potential (LRP) [55], [56]. The RP can be recorded up to two seconds before the movement's onset and is pronounced at central electrode sites [57]. The LRP has, in case of arm and hand movements, its maximal amplitude contralateral to the side of movement above sensorimotor areas of the brain and will occur just before movement onset. By detecting brain patterns by BR that are related to movement preparation processes, the onset of movement can be inferred by eBR and used to adapt the interface, i.e., exoskeleton, for an easier lock-out from a rest situation (PC mode in Fig. 3). However, even if BR detects movement intention, the exoskeleton's mode is not directly changed. Any change from PC to a FM mode will only happen after the inferred movement onset is confirmed by the force sensors that are integrated in the exoskeleton (see Fig. 3 and Video S3). This prohibits faulty behavior of the exoskeleton but improves interaction by reducing the force that is required for lock-out in case the inferred behavior is indeed executed [52].

### Goals: Applying and Improving BR during Complex Interaction

Although we were able to show in the teleoperation scenario that our approach of adapting both HMIs by eBR works online and improves interaction [49], [53], it is not clear whether or not our approach of BR to relate brain patterns that were detected by means of machine learning methods to certain states of the human is appropriate for complex human-machine interaction scenarios. In the given application example the intentional state “movement intention” and the cognitive states “recognition of important stimuli and task coordination” are expected to be accompanied by ERPs, like the RP and LRP for “movement intention” and the P300 and prospective positivity for “recognition of important stimuli and task coordination” as explained above. To support that BR indeed allows to detect these states, correlation between brain patterns detected by ML and the above mentioned ERP activities must be shown. One has to point out that other brain activity besides the expected ERP activity will be learned by the classifier especially since applied ML methods can make use of all available signals from all electrodes. This may on one hand decrease classification performance since the classifier might learn unstable features that are for example present during training but not during testing and might reduce the reliability of inferred behavior by eBR since brain processes other than the assumed ones might evoke the brain patterns that were detected by BR. On the other hand, other brain activity than the here investigated one will surely contribute positively to the classification performance. Hence, our goal was not to prove that evoked brain activity not investigated here is not involved. Rather, the goal of this work was to support the application of BR in complex human-machine interaction scenarios. Therefore, we investigated whether predictions made by ML based on detected brain patterns can be related to known patterns in the EEG, here ERPs, that are well understood in their meaning with respect to the brain's functioning as well as their psychological effects. Since we wanted to perform the above explained investigations during complex interaction, the chosen experimental setups had to cover certain aspects of the teleoperation scenario. Two experimental scenarios were designed. The teleoperation scenario described above was not used since an investigation in this scenario with a high amount of subjects is quite-time consuming and experiments could not easily be repeated and reproduced. Moreover, since in the teleoperation scenario two different applications for eBR were implemented in the second scenario we investigated a *dual* BR approach.

We further systematically investigate experimentally the performance of BR with respect to training data. To improve performance of BR by choosing most appropriate training data (here the relevant training window as well as combinations of training windows) is important since BR as a passive approach cannot make use of *direct feedback* during training to optimize brain activity as it is common for the application of active and reactive BCIs and will hence not profit from effects of biofeedback [12], [58]. Even more critical than this is the fact that a complex application may not produce enough training data while performance of ML strongly depends on the amount and quality of the training samples. Our robotic application uses BR in situations which occur irregularly and are hard to reproduce for training. One way to deal with this issue is to substitute the underrepresented training class by a training class for which more and similar examples can be acquired. Such approaches are already applied with success. In [59] an overview is given when and how transfer learning can be applied in general. For the detection of brain patterns, classifier transfer was also proposed. Observation error related potentials (ErrPs) were detected in a task on which the applied classifier was not trained [60]. In this study a classifier for the same type of ErrP (observation ErrPs) was transferred between tasks. In [61] we showed that a classifier which is trained on one type of ErrP can classify another type of ErrP. Although the underlying kind of interaction (active versus passive interaction) is different, one can assume that similar brain processes are responsible for the detection of errors. In this work we want to investigate whether it is possible to transfer a classifier between classes used for training and testing that are similar with respect to the fact that the individual ERPs do not contain a specific component, i.e., a P300. Our hypothesis is that this is possible, if ERP analysis shows that the relevant component, i.e., the P300, is missing in both cases. Hence, for classifier transfer we propose that the classifier does not have to be trained and tested on examples that are evoked by the same brain processes (like error detection processes as explained above), but by brain processes, which might be different, but evoke brain patterns, which are similar in shape and characteristics, i.e., miss a prominent ERP component.

To summarize, in the following we will present results of two studies, which show that BR can be applied during complex human-machine interaction to detect patterns in the EEG in *single trial* with a high accuracy. In Part “Labyrinth Oddball Scenario – Recognition of Important Stimuli and Task Coordination Processes” we investigate, whether the cognitive states “recognition of important stimuli and task coordination” can be correlated to the results of ML analysis. For this goal the EEG was analyzed by averaged ERP analysis and single trial ML analysis. The applicability of classifier transfer between different classes is investigated in Sec. “Window of Interest and Transferability of Classifier”. Furthermore, we present results on improving the detection accuracy by choosing optimal training windows based on ERP and ML analysis, i.e., show how to optimally combine different training windows (see Sec. “Window of Interest and Transferability of Classifier” and Sec. “Combination of Training Windows for a Robust Detection of Movement Intention” in Part “Dual BR Scenario Armrest – Simultaneous Detection of Two States”). In Part “Dual BR Scenario Armrest – Simultaneous Detection of Two States” we further present results for detecting both the intentional state “movement intention” as well as the cognitive state “recognition of important stimuli and task coordination” within one experimental setup. Such a dual BR approach that enables the simultaneous detection of two different brain states is an important requirement to enable eBR to adapt two HMIs, i.e., the OMS and an exoskeleton, within one application, i.e., the above described teleoperation scenario. Furthermore, in Sec. “Performance of BR in the Detection of a Highly Underrepresented State” we replicated some results of the first study under more realistic conditions to confirm that our approach of classifier transfer works even in case of reduced numbers of samples of the relevant class. In Sec. “Conclusions” conclusions are drawn regarding the results gained in our studies with respect to the applicability of BR for *self-controlled, predictive* HMIs in robotics.

## Labyrinth Oddball Scenario – Recognition of Important Stimuli and Task Coordination Processes

**Figure 4:**
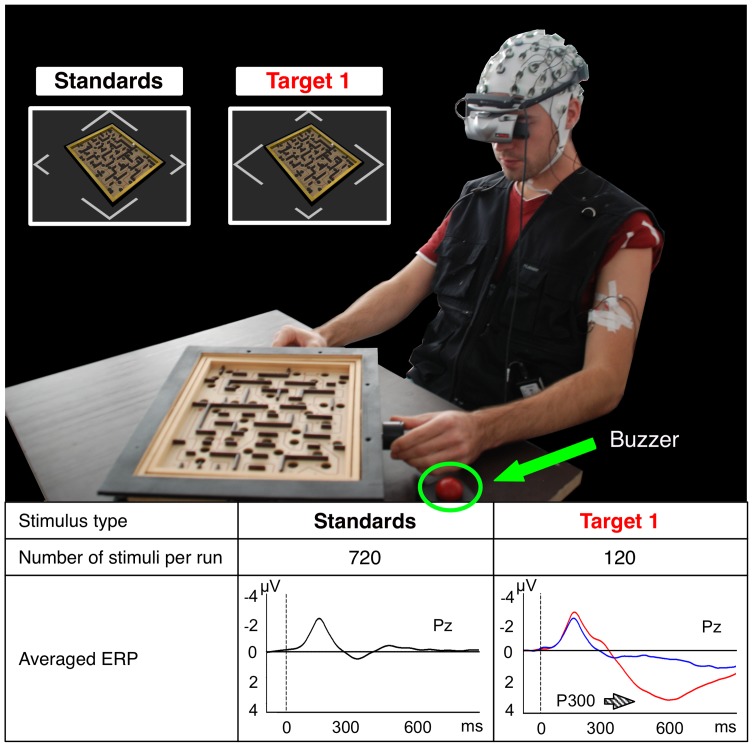
Experimental setup Labyrinth Oddball. In the Labyrinth Oddball setup subjects perform a dual-task, i.e., they play a virtualized labyrinth game and react to less frequent first and second target stimuli by pressing a buzzer. A second target is presented in case that the first target was missed. Brain activity recorded after the different stimuli was averaged over all subjects, sessions, and runs (total number of trials after artifact removal: *target 1* (red ERP curve, right side): 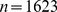; *missed target 1* (blue ERP curve, right side): 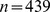; *standards* (black ERP curve): 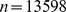).

To support our hypotheses that ERP activities evoked by the presentation and processing of different stimuli contributes strongly to the separability of classes in ML analysis and that BR can hence be applied to detect the cognitive states of “recognition of important stimuli and task coordination” the test scenario *Labyrinth Oddball* (Fig. 4) was developed. By means of this test scenario we further show that classifier transfer is possible between classes that contain examples of similar shape and characteristics, i.e., miss a P300. The scenario allows to investigate the EEG activity of an operator who is controlling a device while reacting to incoming infrequent information at the same time. This mimics the situation in the described teleoperation scenario (see Fig. 1), where the operator performs a main task involving continuous motor activity (telecontrol of the robot), while monitoring and responding to important information that is given to him. In the teleoperation scenario, response time is expected to jitter in a wide range depending on the workload that is induced by the main task. This is expected to be similar in the Labyrinth Oddball setup (for visualization of the scenario see Video S4).

The Labyrinth Oddball scenario can be described as follows: A subject plays a virtualized BRIO® labyrinth game wearing a head mounted display (HMD). This demanding task was chosen to put the subject into a situation of high workload while performing the second task, which is to react to infrequent warnings (first and second *target* stimuli, see “Target 1” in Fig. 4 for first targets, second targets were represented as a full form in the shape of a diamond matching the color and size of the first target) by pressing a buzzer. Subjects were asked to respond immediately and not to ignore any target stimulus. Target stimuli (infrequent, important information) were mixed up with *standard* stimuli (frequent, unimportant information that require no response, see “Standards” in Fig. 4; the corner with the longer sides points upwards instead of sidewards if compared with the first targets) in a ratio of about 1∶6. The inter-stimulus interval (ISI) was 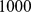 ms with a random jitter of 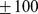 ms. For more general details about this experimental setup see [44]. Since the manipulation task was very demanding, a rather long response time from 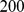 ms to approximately 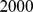 ms (i.e., 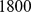 ms to 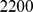 ms due to jitter in inter stimulus interval) after target stimulus presentation was allowed during the recording of training data before a second warning was presented. In case there was no response within this period, the trial was labeled as *missed target*. On the second target a response time of 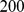 ms to 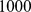 ms was allowed. In contrast to the scenario used in [44], visual presentation (shape and color) of standard stimuli that require no response and first target stimuli that require a response were kept very similar (see Fig. 4) in order to avoid differences in early visual processing of the stimuli. This assures that differences in the EEG recorded after the presentation of both stimuli types were mainly caused by processes of higher cognitive processing.

**Figure 5:**
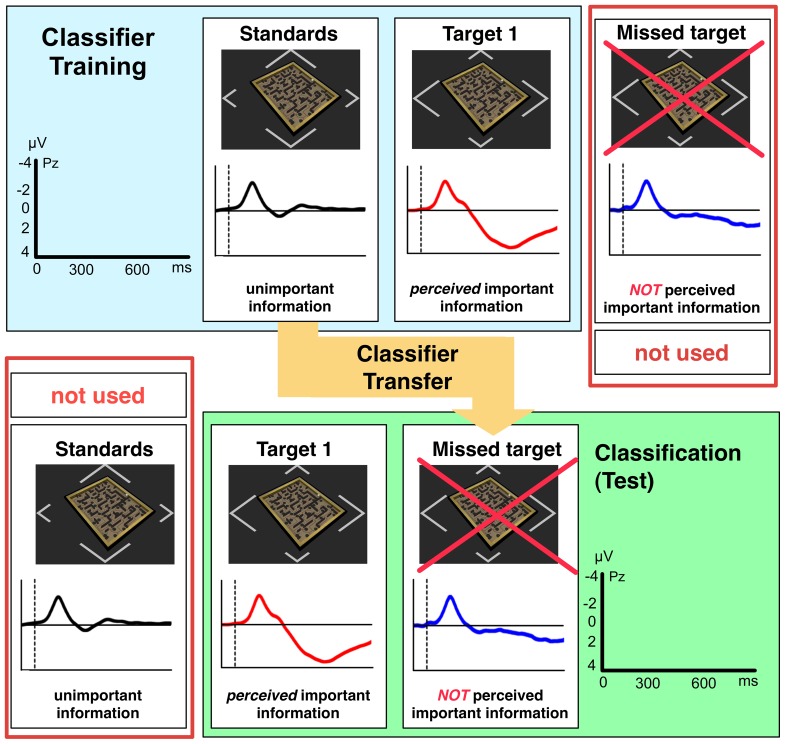
Classifier Transfer. Transfer of classifier between classes is visualized. Classifier transfer is applied between the class *standard* and *missed targets*. Hence, for training the class *standard* was used, in test the class *missed targets* was used instead.

As discussed in Sec. “Introduction” classifier transfer is possible between two classes if the patterns of the samples of both classes (used for training and for test) are similar in shape and characteristics. For the detection of target recognition processes by BR we substituted our test class in ML analysis, i.e., infrequent samples evoked by situations in which the user missed the first targets (*missed targets*), with a training class of frequent samples (*standards*), i.e., EEG instances evoked by frequent unimportant information to which the user was not required to respond (Fig. 5). This approach was based on the assumption that ERP activity evoked by standards is very similar in shape and characteristic to ERP activity evoked by missed targets and that both differ from ERP activity evoked by targets, which represent the second training and test class. The expected similarities between EEG activity evoked by standards and missed targets is mainly the absence of a P300. Only perceived target stimuli will evoke a P300 (mainly P3b due to the task relevance of the target stimuli). Our hypothesis is that the P300 substantially contribute to the class separability in ML learning. We further assume that the *absence* of target detection processes, either because of a failure of recognition or complete miss (for missed targets) or because it is not required (for standards) mainly contributes to the similarities between ERPs evoked by standards and missed targets.

Another implicit difference is that response behavior is only executed after *target* stimuli and not after *standard* and *missed target* stimuli. It is known that motor-related potentials that are evoked by the preparation and execution of response behavior can influence the amplitude of P300 (i.e., P3b in [43]) which is expected to be evoked by targets but not by standards and missed targets. However, we can largely rule out a major impact on P300 amplitude differences by motor-related activity and by this a major influence of response preparation and execution on class separability and classifier transferability in our experiment for at least three reasons: First, studies showed that the P300 latency is not correlated with reaction time [42]. Only in case that a very fast response time is requested a correlation can be found between response preparation and P300, i.e., P3b (see [43] for discussion). In the *Labyrinth Oddball* scenario we expected that motor response activity will be late and poorly time-locked to the stimulus onset with a low correlation due to the dual task condition. Hence, related EEG activity is not expected to overlap largely. Second, possible differences related to motor activity are most prominent at frontal and central electrodes [57] and should not heavily influence ERPs at electrode Pz, where highest amplitudes for P3b are expected. Third, subjects are constantly performing the labyrinth task during the experiment and therefore motor-related activity (not corresponding to the oddball response) is evoked while processing all types of stimuli. Thus, motor activity is not only prominent after *target* stimuli. Furthermore, it could be shown that the execution of button press in a simple oddball setting does reduce the amplitude of the midline P3b [43]. By weakening the amplitude of the P3b by motor response on *targets*, ERP activity that is evoked by *target* stimuli would be more similar to ERP activity on *missed targets* than the latter to *standards* which is rather the opposite of the hypothesis we want to validate here.

To support our hypothesis, we conducted an ERP study in the *Labyrinth Oddball* scenario investigating differences in ERPs after the presentation of *standard*, *target* and *missed target* stimuli. First, the behavior of the subjects is analyzed to differentiate between EEG trials with correct, incorrect and missed behavior. Further, the reaction time for correct trials is calculated. In the average ERP analysis we focus on EEG activity occurring 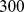 ms after stimulus onset at electrode locations Cz, Pz, and Oz, since the P3b component should be expressed at that time or later with maximal amplitude at electrode positions Cz and/or Pz in case of target recognition [41]. The relevance of the P300, i.e., P3b, for class separability and classifier transferability is investigated by the above mentioned average ERP analysis and by a systematic machine learning (ML) analysis. By comparing the results of both analyses we investigate whether ERP activity recorded in the time range of the P3b is suited to make predictions on the transferability of a classifier. In the ML analysis we systematically train a classifier on different sub-windows to evaluate how well the transfer works for different windows. Following and depending on the outcome of the ERP average study we investigate which window and which window size is most important and what performance can be achieved after optimization of preprocessing and classification. A reduction of window size contributes to lower computational costs and is therefore desirable for online analysis.

### Methods

#### Experimental Procedures and Data Acquisition

Six subjects (males; mean age 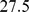, standard deviation 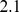; right-handed, and normal or corrected-to-normal vision) took part in the experiments. Subjects were instructed to respond to all *target* stimuli even in case they were uncertain. By this procedure, we ensured that *missed targets* were indeed missed and not perceived as important and task relevant stimuli. Subjects were in a competition to miss as few as possible *targets* while achieving good performance in the game. Recognizing and responding to all targets was rated higher than performing the senso-motor, i.e., labyrinth task. One subject had to be excluded in retrospect due to extensive eye blinks which made average ERP analysis impossible. The experiment was split into two sessions with at least one day rest in between. In each session, each subject performed 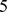 runs with 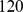 *target 1* stimuli (important information) and about 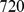 *standard* stimuli (unimportant information, shape of stimuli see Fig. 4). Stimuli were presented in random order.

While the subjects were performing the task, the EEG was recorded continuously (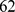 electrodes, extended 10–20 system with reference at FCz) using a 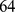 channel actiCap system (Brain Products GmbH, Munich, Germany). Two electrodes of the 64 channel system were used to record the electromyogram (EMG) of muscles of the upper arm (M. bizeps brachii) related to the buzzer press in order to monitor muscle activity. Impedance was kept below 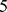 k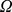. EEG and EMG signals were sampled at 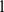 kHz, amplified by two 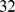 channel BrainAmp DC amplifiers (Brain Products GmbH, Munich, Germany) and filtered with a low cut-off of 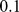 Hz and high cut-off of 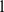 kHz.

#### Ethics Statement

The study has been conducted in accordance with the Declaration of Helsinki and approved with written consent by the ethics committee of the University of Bremen. Subjects have given informed and written consent to participate.

#### Behavior

For behavioral analysis we investigated the performance of the subjects in the oddball task. For this, we analyzed the subject's correct behavior and incorrect behavior (commission error, i.e., response on standard stimuli and omission error, i.e., missing response on target stimuli).

**Figure 6:**
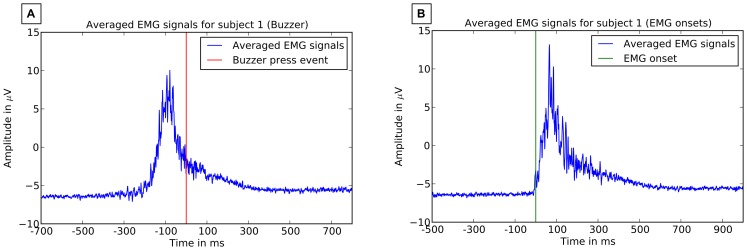
Averaged EMG activity. Average EMG activity of subject 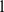 that was averaged based on two different events is displayed. A: Averaged activity based on buzzer press event is shown. B: Average activity based on EMG onset is shown.

Further, we investigated the response times and jitter in response times based on buzzer events and EMG onsets (see Fig. 6 for averaged EMG activity based on EMG onset and buzzer event). The onsets in the EMG signal had to be labeled manually, due to poor signal quality and constant movement of the subject an automated onset detection as described in [62] was not possible. For the analysis of EMG onset the signals from the two unipolar EMG channels were subtracted from each other to calculate a bipolar signal. The raw bipolar signal was preprocessed using a variance based filter with a window length of 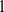 s [62]. The resulting signals were visually inspected and each onset was marked in the EEG data. The single response time was then measured as interval between the target onset and the corresponding EMG onset. Single response times on the buzzer events were measured as time between the onset of stimulus presentation and the onset of buzzer press. Further, we calculated the median of response time over all sets (3 sets 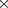
2 sessions) for each subject and also minimal response time and maximal response time. After that, the mean of subject's medians was calculated.

#### Average ERP Analysis

To identify relevant ERP activity an average ERP analysis was performed. EEGs from runs 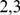 and 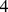 of both sessions were analyzed off-line with the BrainVision Analyser Software Version 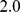 (Brain Products GmbH, Munich, Germany). Run 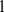 and 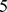 were not used for analysis to reduce the amount of data and thus processing time for the ML analysis presented in Sec. “ML Analysis”. We chose the middle runs to minimize side effects due to training or exhaustion.

EEGs were re-referenced to an average reference (excluding electrodes Fp1, Fp2, F1, F2, PO9, PO10, FT7–FT10 due to artifacts and electrodes TP7 and TP8 which were used to record EMG activity) and filtered (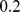 Hz low cutoff, 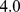 Hz high cutoff). The low-pass filter was chosen with an untypical low cutoff frequency, since results of average ERP analysis should be compared with results of ML analysis. Although different pass bands are reported in P300 classification (see [63], [64]) a study about the important factors on P300 detection concluded that the main energy of this type of ERP is concentrated below 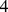 Hz [64]. Our own investigations support this conclusion (see for example [65]). An ERP analysis of EEG data from a very similar experimental setting which considers a wider frequency range (higher low pass filter) is currently under preparation. Preliminary results are published in [47]. Artifacts (e.g., eye movement, blinks, muscle artifacts, etc.) were rejected semi-manually (maximal amplitude difference in 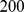 ms intervals was 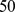 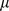
V, gradient 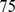 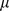
V/ms, low activity was 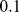 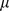
V over 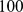 ms). EEGs were segmented into epochs from 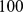 ms before to 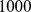 ms after stimulus onset. Epochs were averaged separately for each stimulus type. Only segments in which a stimulus of type target was followed by a response within the given response time contributed to mean ERP curves on the stimulus type *target*. Segments in which no response followed after a stimulus of type target were defined as missed target trials and contributed to generate mean ERP curves on the stimulus type *missed target*. Baseline correction was performed before averaging (pre-stimulus interval: 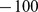 to 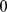 ms). In case of missed target events a second target (target 2) followed. In this study we did not evaluate ERP activity evoked by stimulus type target 2 and missed target 2.

Amplitude differences were analyzed using repeated measures ANOVA with the within-subjects factors *stimulus type*, *electrode*, and *time window* and between-subjects factor *subject*. To find the expected P300 effect, we compared amplitude differences between the three stimulus types (standards, targets, and missed targets). Additionally, the factor *electrode* (Cz, Pz and Oz) served to investigate spatial differences in the P300 effect. *Time window* was used as factor, since visual inspection of the averages of activity evoked by targets revealed multiple peaks in the time range of 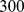
–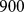 ms for each subject. Therefore, we divided the 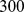
–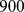 ms window into two separate windows (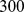
–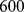 ms and 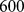
–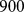 ms after the stimulus) to cover early and late parts of the broad peak (as seen in grand average in Fig. 4), accounting for multiple, possibly overlapping positive ERP components. To investigate subject-specificity of the effects, *subject* was used as a between-subjects factor. Where necessary, the Greenhouse–Geisser correction was applied and the corrected 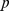-value is reported. For pairwise comparisons, the Bonferroni correction was applied.

#### ML Analysis

All ML evaluations have been performed using the open source signal processing and classification environment pySPACE [66]. Data processing was as follows: Windowing and preprocessing were performed directly on the raw data from the recording device. In order to avoid that preprocessing artifacts such as, e.g., filter border artifacts, influence classification performance, we performed the complete preprocessing (including decimation and filtering) on a larger window between 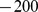 and 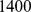 ms relative to the stimulus onset. We chose the following preprocessing based on the rationale issued above (see Sec. “Average ERP Analysis” and [64]): The data were baseline-corrected (with 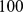 ms window prior to stimulus onset), decimated to 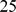 Hz and subsequently lowpass filtered with a cut-off frequency of 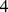 Hz.

As in the ERP analysis, run numbers 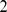, 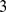 and 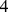 of both sessions were used for training and testing. In contrast to the average ERP analysis described above we included the early time window of 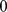
–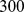 ms in the ML analysis. This was done to control for the fact that early time windows may still contribute to the classification of the different classes (*standards*, *targets*, *missed targets*) even though we hypothesized that main differences are caused by the P300 effect (see Sec. “ Relevant Averaged ERP Activity”).

It is important to note here that several dependencies have to be kept in mind when evaluating the results: First, performance depends on window size since a larger window contains more features and thus a higher dimensionality of the signal. Second, the classifier parameters depend on the underlying data (and dimensionality). However, the purpose of this investigation was to compare different windows (and sizes) concerning their quality for classification, so we assessed the results always with respect to window size and starting point of the window and performed statistical analysis only on windows of equal length.

Furthermore, the parameters of the classifier were adjusted to an unspecified value to omit data-dependent effects: in the entire analysis, we used a support vector machine (SVM) as implemented in *LIBSVM* [67] (SVC-C with a linear kernel) with a fixed complexity of 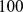 simulating a hard margin. Hence, we cut different windows by varying starting point (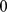 ms-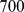 ms) and window size (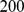 ms-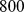 ms) in steps of 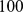 ms. Data used for training and testing were different, as outlined above: We trained on *standards* and *targets* of one experimental run and tested *missed targets* versus *targets* of another run within one session. All possible combinations of the above mentioned runs within one session were tested. Classifier features were the preprocessed time-channel values, i.e., the amplitudes.

The corresponding classification performance was computed using the area under curve (AUC) [68] which is an indicator of general separability of the two classes in the data. AUC is the area under the receiver operating characteristics curve. This curve maps the different true positive rates (TPR) and false positive rates (1-TNR) obtained when the decision boundary is varied from 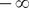 to 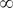. The AUC is then computed as the integral of the resulting function. In this way, we investigated the linear separability of the data essentially independent of the applied classifier.

For statistical inference, we chose three time windows from the aforementioned temporal segmentation that match the later time windows which had been chosen for ERP analysis (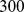
–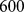 ms, and 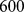
–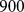 ms see Sec. “Average ERP Analysis”) and the early time window of 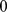
–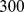 ms. This procedure relates the results of the classifier performance-based approach to the results of the ERP analysis. Classification performances for the different window sizes were statistically analyzed using repeated measures ANOVA with the within-subjects factors *time window* (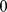
–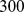 ms, 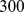
–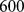 ms, and 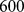
–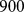 ms) and *subject*. Corrections were applied where necessary. Classification performance after optimizing the classifier were analyzed using repeated measures ANOVA with *subject* as within-subjects factor. Where necessary, the Greenhouse–Geisser correction was applied and the corrected 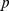-value is reported. For multiple comparisons, the Bonferroni correction was applied.

In a further analysis we investigated the possibility to improve classification performance by the combination of information from two windows. We combined the middle time window (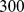 to 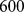 ms) with both other time windows (early: 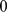 to 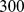 ms and late: 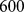 to 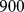 ms time window) separately.

To determine classification performance that can be achieved under optimized conditions we finally performed a final analysis with the goal to get a better estimate of the applicability of our approach of classifier transfer between the classes *standard* and *missed target*. The processing window was chosen based on the results of the systematic ML analysis explained above. We performed a classifier optimization of the SVM parameter complexity using a 5-fold cross validation in combination with a pattern search algorithm [69] to evaluate the overall performance in the application with an adjusted classifier.

In this subsequent investigation we used an optimized SVM on a chosen time window. Further, we computed the balanced accuracy (BA) as a performance measure for the chosen time window. The balanced accuracy [70] is the arithmetic mean of true positive rate (TPR) and true negative rate (TNR) and calculated accordingly
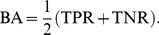
(1)


Both performance measures used (AUC and BA) are insensitive to unbalanced or even changing ratios of the two classes (positive class P and negative class N), which is most important in the application where we have an oddball-like situation with frequent and infrequent examples. It holds for both metrics that a value of 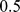 means guessing and 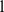 means perfect classification.

### Results

#### Behavior

In total 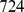 omission errors (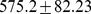) occurred, thus 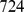 *missed targets* were observed and 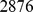 *targets* stimuli were found with correct responses. No commission error (i.e., responses on *standards* stimuli) could be found.

**Figure 7:**
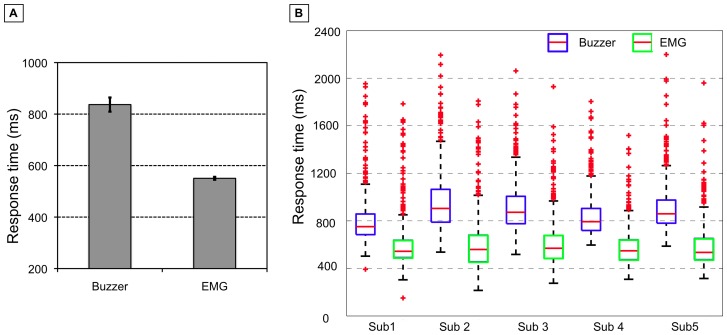
Evaluation of response time. The mean and median of response time for each subject across two sessions based on EMG and buzzer press events are displayed. A: Mean of response time. B: Median of response time.

Figure 7 shows the median response time for each subject across two sessions. Based on the buzzer press event, responses occurred 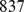 ms after the target stimuli (mean of subject's medians). The median of minimal response time was 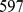 ms and the median of maximal response time was 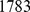 ms. The difference between the minimal and maximal response time was between 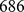 ms and 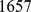 ms (median: 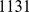 ms). EMG onsets began even earlier in time (mean of subject's medians: 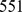 ms). The median of minimal response time was 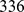 ms and the median of maximal response time was 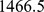 ms. The difference between the minimal and maximal response time was between 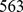 ms and 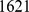 ms (median: 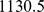 ms). No difference exists between median difference in response time based on the buzzer event (median: 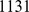 ms) and median difference of response time based on the EMG onset (median: 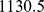 ms).

#### Relevant Averaged ERP Activity

**Figure 8:**
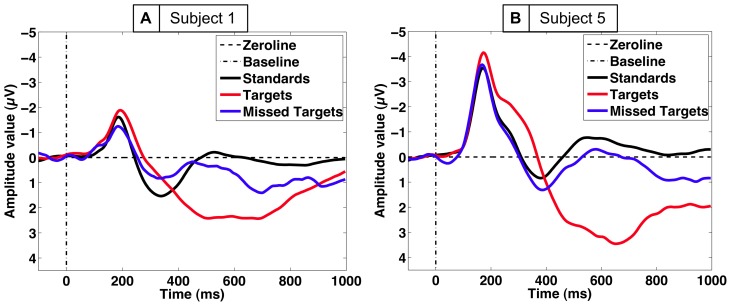
Averaged ERPs in the Labyrinth Oddball scenario. Different averaged ERP patterns evoked by standards, targets, and missed targets are shown for two subjects. A: Subject 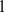: No significant difference in ERP amplitude between targets and missed targets but significant difference in ERP amplitude between standards and missed targets for the late window was found. B: Subject 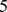: A higher P300 effect on targets compared to both standards and missed targets and no significant difference in ERP amplitude between standards and missed targets for the late window was found.

The grand average over all subjects of the standard, target and missed target ERP pattern in the centro-parietal electrode (Pz) is depicted in Fig. 4. Significant differences between standards and targets (i.e., P300 effect) were observed [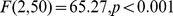, pairwise comparisons: standards vs. targets: 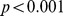]. The P300 effect was stronger at the electrodes Cz and Pz compared to the electrode Oz [P300 effect at Cz: 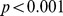, P300 effect at Pz: 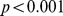, P300 effect at Oz: 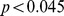]. The significant amplitude difference between the ERPs evoked by targets and missed targets stems from a higher positive amplitude on targets for both time windows [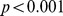]. This higher positivity elicited by targets was significant for four subjects [targets vs. missed targets: 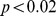 for four subjects, 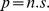 for one subject (subject 1), see Fig. 8]. Furthermore, no subject showed differences between ERPs evoked by missed targets and standards in the 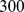
–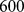 ms time range recorded over central electrodes [standards vs. missed targets: 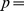 n.s.]. However, in the 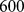
–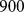 ms window, amplitude differences between missed targets and standards are more subject-specific [standards vs. missed targets: 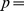 n.s. for subject 4 and 5, 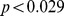 for subject 1, 2, and 3, see Fig. 8].

To summarize, a P300 effect elicited by targets was observed for both time windows and in all subjects with a maximum amplitude intensity at the central and parietal electrodes (Cz and Pz). The morphology of the ERP form elicited by missed targets is, especially in the 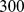
–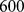 ms time window, similar to ERP forms elicited by standards and supports our hypothesis that EEG instances evoked by standard stimuli can potentially be used to substitute EEG instances evoked by missed targets during training. For the later time window results differed. Only two subjects showed no differences between standards and missed targets.

#### Window of Interest and Transferability of Classifier

**Figure 9:**
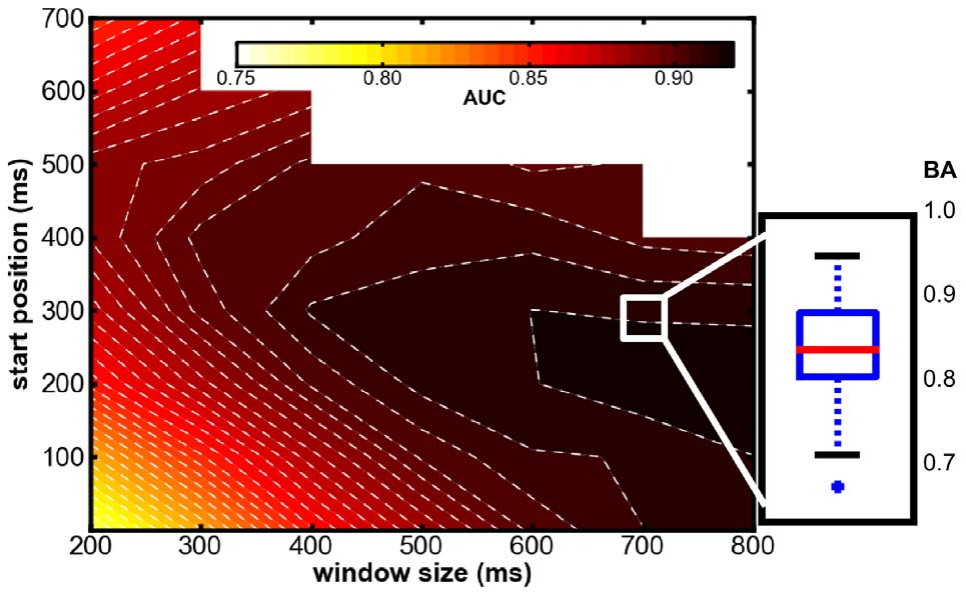
Classification performance obtained in the Labyrinth Oddball scenario for different windows of EEG data. The dependency between classification performance and window size as well as start point of window are displayed for the classification of missed targets versus recognized targets. The start position (y-axis) is given relative to stimulus onset. The inset on the right indicates the optimized performance using the window from 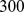 to 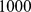 ms. The different windows are compared using the AUC, while the optimized performance is given as BA.

The results in Fig. 9 illustrate how the separability of the two classes *missed targets* versus *targets* varies when different time windows are used for classification. For small and early windows (before around 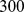 ms) the performance is lowest but above random guessing. For small window sizes (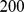
–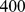 ms) the performance reached a maximum when used with windows starting after 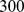 ms. With increasing window size performance also increases, which is yet impacted with the increased dimensionality of the data (more dimensions imply more information for the classifier) and has therefore to be considered carefully.

**Figure 10:**
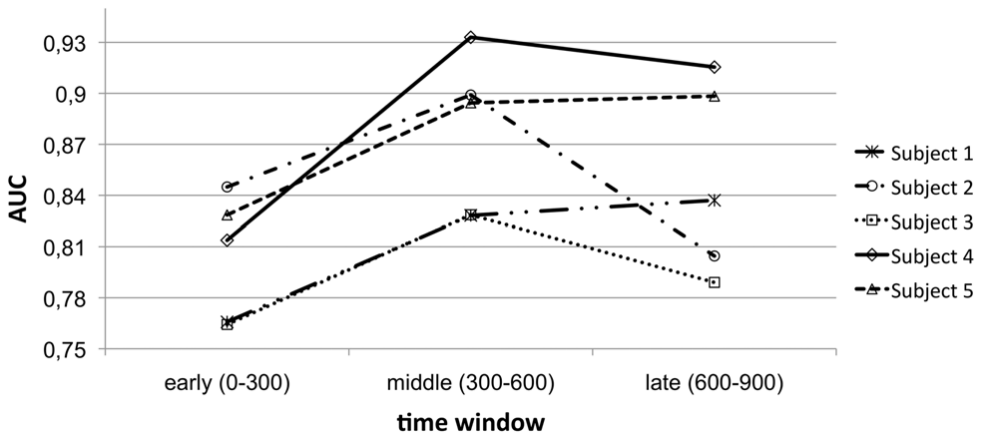
Classification performance for different time windows. The mean classification performance is shown for each time window and each subject.

To investigate the amount of information in each time range, we compared performances on training data with fixed window sizes of 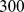 ms as illustrated in Fig. 10. The statistical analysis of the AUC values shows that performance is clearly affected by the choice of the time window [main effect of *time window*: 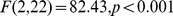] and that classification of the middle window (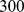 ms–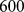 ms) and the late window (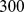 ms–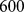 ms) clearly yields higher performance compared to the early window (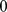 ms–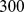 ms) [early window: mean AUC of 0.82, middle window: mean AUC of 0.90, late window: mean AUC of 0.88, multiple comparisons: 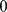
–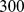 ms vs. 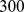
–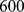 ms: 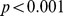, 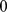
–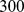 ms vs. 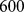
–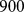 ms, 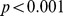, 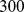
–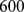 ms vs. 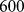
–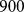 ms: 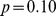].

In the further analysis were we combined the middle time window (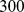 to 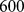 ms) that is showing the highest classification performance for most subjects (see Fig. 10) with both other time windows (early: 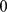 to 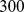 ms and late: 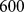 to 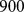 ms time window) separately, the main result was that classification performance could be improved by the combination of the *middle* and *late* time window compared to the combination of the middle and early time window [main effect of *combined time window*: 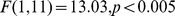, combination of the middle and early window: Mean of AUC of 0.89, combination of the middle and late window: Mean of AUC of 0.92, pairwise comparison: combination of the middle and early window vs. combination of the middle and late window: 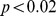] again supporting our hypothesis that later cognitive activity is most important for the prediction of the success of cognitive processing.

**Figure 11:**
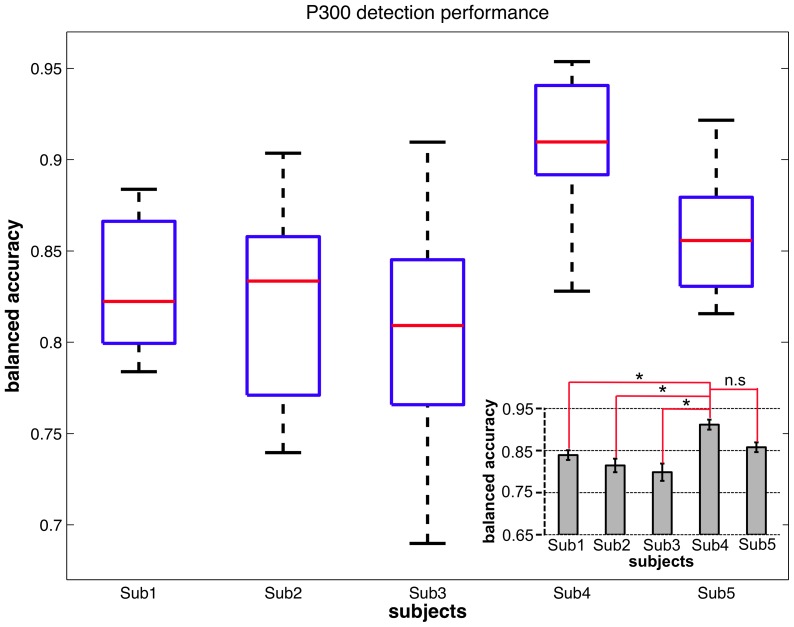
Classification performance in the Labyrinth Oddball scenario. For each subject for a window from 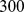 to 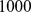 ms the evaluated classification performance and statistics are shown. The red lines in the main diagram mark the median values of obtained classification performances for each subject. The inserted diagram shows that highest classification performance was obtained for subject 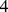 and 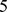 (mean classification performance and standard error of mean (SEM) are depicted).

Given the results presented above we obtained the best results when starting the windows 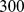 ms after the stimulus was presented (depicted in Fig. 9). This supports our hypothesis that P300 related processes contribute substantially to class separability. Based on theses findings, we decided to use a processing window in the time range between 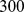 ms and 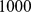 ms. As described in the methods section, we now used an optimized preprocessing procedure and classifier for this window. On average, a BA of 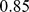 (standard deviation: 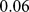) was obtained. While the measure of the AUC served for finding the interesting window ranges, this performance measure now reflects what the particular classifier is able to achieve. The distribution of the results is illustrated in the inset in Fig. 9 and the classification performance for each subject is depicted in Fig. 11. A significantly higher classification performance compared to all other subjects (except for subject 5) was shown for subject 4 [main effect of *subject*: 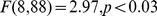, details see Fig. 11 lower right].

It is worth to point out that average ERP analysis for subjects 4 and 5 (with better classification performance) in contrast to all other subjects could not reveal any significant differences in amplitude of averaged ERP forms evoked by *standards* and *missed targets* in both time windows (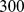 to 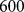 ms and 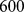 to 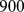 ms). Based on our hypotheses, such similarity between ERP forms evoked by *standard* and *missed target* stimuli and a clear absence of P300 and later EPR activity that may be related to task coordination would suggest a good outcome for classifier transfer and high performance as was shown here. Hence, results of ERP analysis can under certain conditions be used to infer classification performance.

### Discussion: Labyrinth Oddball Scenario

Results of ERP and ML analysis confirm that ERPs evoked by stimulus recognition and subsequent processes, e.g., change of task and preparation of response, are most important to detect the state of target recognition by BR. This is a basic prerequisite for eBR to infer response behavior of the operator. We showed that a classifier trained on the classes *standards* versus *targets* can be successfully transferred to classify the classes *missed targets* versus *targets*. Results of ERP analysis of ERPs evoked in the middle time window that were found to be maximally expressed on central and parietal electrodes (Cz and Pz) were used to infer on classification performance. Thus, it is likely that the signal that is maximally expressed at these electrodes contributes most to the differences and similarities of the overall signal on all three types of stimuli.

Our hypothesis that ERP activity evoked by unimportant *standard* stimuli is similar in shape and characteristic to ERP activity evoked by important stimuli that were not recognized as such (*missed targets*) was supported by the results. Further, our results indicate that this similarity is in the middle time windows mainly caused by the absence of target recognition processes, since the P300 is either missing or massively reduced in amplitude. Certainly, processes later than the evaluation and classification of stimuli (evoking a P300) that are related to task set preparation or response preparation and execution will also be involved [47]. For example, for some of the subjects ERP activity evoked by unimportant standard stimuli and by missed target stimuli shows significant differences in the later time window which may be related to late task set preparation processes [46] or late P300 activity that did not lead to a successful stimulus evaluation as discussed in [42] and requires further investigation (see Fig. 8, e.g., subject 1). Although a prominent similarity between *standards* and *missed targets* is the missing of a response of the subject, our results show that response related activity should not have a major influence on transferability of the classifier, since response time to individual target stimuli does widely vary (see Sec. “Behavior”).

Results of ML analysis finally show that early stimulus processing in the time window 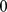
–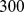 ms was not equally important as EEG activity in the later time range (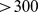 ms) investigated here. However, early brain activity contributed as well. This might be caused by differences in attentional processes which have to be investigated in future experiments and analysis.

To summarize, from our results we conclude that brain activity evoked by infrequent, unimportant stimuli (*standards*) in the investigated low frequency range is highly similar to brain activity evoked by *missed targets*, which are important stimuli that were not successfully processed, i.e., not recognized as important stimuli or completely missed. To substitute infrequent examples of the class *missed targets* by frequent examples of the class *standards* during training is possible and supports our hypothesis that transfer between classes is a feasible approach for applying BR in scenarios in which the amount of training data is way too small to implement methods that can handle few training data [71]–[73] (for a brief discussion see [74]). Hence, the problem of few training examples in realistic scenarios can be solved by our approach of classifier transfer with a high classification performance, and can be improved by choosing appropriate window combinations. The choice of window, samples used for transfer and the combinations of windows were first defined by knowledge about underlying brain activity gained from average ERP analysis and confirmed by systematic ML analysis. Hence, it is shown that average ERP analysis can be a useful method to choose appropriate training data, especially if processes are involved that evoke pronounced patterns in the EEG like the P300.

## Dual BR Scenario Armrest – Simultaneous Detection of Two States

Since the BR system in the teleoperation scenario (see Fig. 1) should not only detect success in the recognition of important information but also movement intention to optimize the exoskeleton's control (see Sec. “Introducation”), a second test scenario, the *Armrest setup*, was developed to test a *dual* BR approach. Experiments were conducted to test whether a simultaneous classification of different brain states is possible by analyzing the EEG recorded in a complex scenario similar to the teleoperation scenario. The Armrest setup copies a realistic dual-task situation that comes closer to the teleoperation scenario than the dual task performed in the *Labyrinth Oddball* scenario (see Part “Labyrinth Oddball Scenario – Recognition of Important Stimuli and Task Coordination Processes”). That is because in the Armrest setup the user is not always able to respond to information (responses to target events were *not* allowed during the rest period – see below) but has to postpone his response. This restriction was most important to prove that our approach still works under realistic conditions in which two motor tasks may influence each other, thus one task inhibits the execution of the other one. Further, it is expected that trained operators of teleoperation scenarios have a low rate of *missed targets*. Hence, to investigate whether it is indeed possible to detect very few instances of missed targets by our approach, we designed a test scenario in which subjects would not miss too many target stimuli.

**Figure 12:**
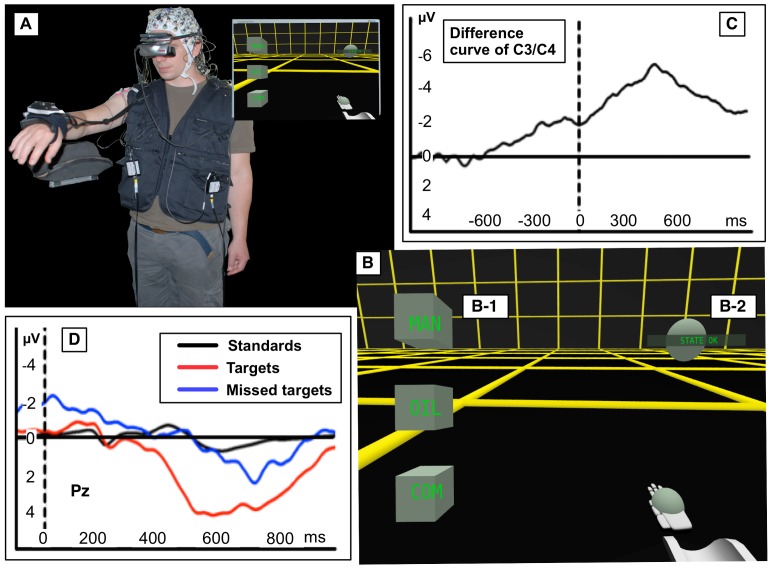
Armrest scenario. The experimental setting *Armrest* is illustrated and most relevant ERP activity evoked by brain processes involved in target recognition and failure in target recognition as well as motor preparation are shown. A: Experimental setup is displayed. B: Three types of virtual response cubes (B-1) and the virtual target ball (B-2) are shown. C: Averaged difference curve between electrodes C3 and C4 (number of trials for movement events: 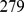) shows differences recorded over the primary motor cortex ipsi- and contralateral to the side of movement (movement onset marker at dashed line). D: Averaged ERP patterns at electrode Pz on different stimulus types (number of *standards*: 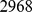, number of *targets*: 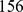, number of *missed targets*: 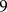) are depicted.

The Armrest setup can be described as follows: Participants of the experiments wore a head-mounted display (HMD) and stood in a dimly lit room while performing a task in a virtual environment. The task was to move the right arm from a rest position in order to reach a virtual target ball which was presented in the upper right corner marking a possible object which could be manipulated in a final application case (Fig. 12A and B). A hand-tracking system was used to detect the point in time when the hand left the armrest. Whenever subjects moved their arm 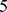 cm away from the rest position, a marker for movement onset was sent and stored together with the EEG (movement marker was set at time point “0”, see Fig. 12C). After entering the target ball (see Fig. 12B–2), the subject returned to the rest position. To support the rest state of the arm, an armrest was designed as part of our testbed. This armrest was integrated into the setup to imitate the strong support of the arm by the exoskeleton during the position control in the teleoperation scenario. The arm and hand of the participant had to stay in the rest position for at least 5 seconds. In case the subject left the rest position too early, the target ball would disappear. This served to avoid too rapid changes between rest and movement which was necessary to assure sufficiently long non-movement periods.

While the subject was performing this task, important versus unimportant information were presented at a ratio of 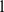
∶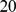. Three different types of important stimuli (target 1) were presented requiring three different responses, namely touching one of three virtual target objects in the virtual scenario as shown in Fig. 12B-1, whereas each kind of warning required touching a particular one. Unimportant stimuli were similar in shape and required no response. In case the user missed an important stimulus (target 1), i.e., did not respond within 10 s after stimulus onset, a second stimulus (target 2), visually highlighted with a different color (orange instead of green), appeared. Again, important stimuli were expected to evoke a P300 and overlapping later ERP components while unimportant stimuli should not (see Fig. 12D). All three virtual response objects were presented at a position in the HMD that followed the head movement to assure that all three objects were always visible on the left side of the visual field. Again subjects were instructed to always respond to the target stimuli. This was the more important task.

In the following we present results that were conducted in the *Armrest setup*. The main goal was to confirm our general approach of BR and to show that it still works in a *dual* BR scenario in which two classified conditions may influence each other. Further, we re-tested the substitution of training examples and hence, partly classifier transfer in this scenario that is similar to the teleoperation scenario and produces even less training examples than the *Labyrinth Oddball* scenario did. Some extra questions were addressed for the detection of movement preparation. During online detection of movement preparation the classifier should to some extent be time shift invariant, hence should not only be able to detect the EEG pattern at the point in time it was trained on, but also at adjacent instances. To obtain such a time shift invariant classifier, Blankertz et al. [75] trained the classifier on two rather than just one window per movement marker. In [54], we systematically analyzed the influence of the number of training windows per movement on classification performance. We found that two training windows significantly improve classification performance. A higher number of training windows per movement does not significantly improve classification. Here, we address the question *which* combination of two training windows (labeled by their end time with respect to the movement marker) provides the best results across subjects. The two windows identified can subsequently serve for all subjects and an exhaustive re-optimization or re-analysis is unnecessary. This is highly relevant for an online application.

### Methods

#### Experimental Procedures and Data Acquisition

Four male subjects (between 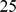 and 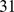 years, right-handed, with normal or corrected-to-normal vision) took part in the experiments which were divided into three runs conducted on the same day. In each run, the subjects had to respond to 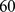 target 1 stimuli. The number of intentional movements from the rest position differed from 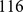 to 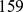. This difference in the number of movement onset trials was caused by the experimental condition that a minimum number for correctly responded target 1 trials was requested per run but the amount of rest periods and their duration (a rest period had to take at least 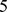 seconds but was allowed to take longer) was not predefined and hence varied between subjects.

For reasons of future data analysis and method development not presented here EEG was continuously recorded with a high density of sensors, i.e., with a 128 electrode system (extended 10–20 system, actiCap, Brain Products GmbH, Munich, Germany), referenced to FCz. Four electrodes of the 128 actiCap system served to record the EMG of muscles of the upper arm (M. biceps brachii and M. triceps brachii) in order to monitor muscle activity. All signals were amplified using four 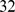-channel BrainAmp DC amplifiers (Brain Products GmbH, Munich, Germany), were digitized with a sampling rate of 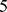 kHz and filtered with a low cutoff of 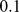 Hz and high cutoff of 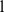 kHz. Impedance was kept below 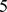 k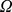.

#### Ethics Statement

The study has been conducted in accordance with the Declaration of Helsinki and approved with written consent by the ethics committee of the University of Bremen. Subjects have given informed and written consent to participate.

#### Behavior

As for the *Labyrinth Oddball* scenario we analyzed subject's performance in the oddball task in terms of the amount of target stimuli with correct response and false reactions (i.e., omission and commission errors) as well as response times and jitter in response time based on the movement marker. We also analyzed how many movements from the rest position were valid, i.e., followed five or more seconds of rest and analyzed the EMG data with the method described earlier for EMG onset detection. Furthermore, the physical movement onset was estimated based on the labels obtained from the interaction of the subject in the virtual scenario and an analysis of movement speed in a study investigating intentional arm movements [62].

#### ML Analysis: Detection of Target Recognition Processes

Data processing for the detection of the relevant patterns in the EEG by ML analysis was performed as for the *Labyrinth Oddball* scenario in the optimized case (see Sec. “Window of Interest and Transferability of Classifier”) using the open source signal processing and classification environment pySPACE [66]. Due to the reduced amount of training examples that could be recorded here, three runs that were recorded in one session for one subject performing the task were joined to a single data set, which was used for performance estimation based on a 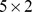-fold cross validation. For performance estimation we had to use a modified cross validation strategy to estimate the classifier's accuracy due to the low number of missed targets. The partitioning of standard and target examples for training as well as the partitioning of target examples for testing was performed as usual to generate mutually exclusive splits, but all missed target examples of the whole dataset were used in every test split for estimating classification performance. Note that due to the classifier transfer the classifier was not trained on the class of missed targets and thus all missed target examples were unknown to the classifier during testing as it also holds true for all targets examples that were used for testing.

To evaluate subject-specific differences in classification performance, the data were analyzed by one-way repeated measures ANOVA with *subject* as within-subjects factor. For multiple comparisons, the Bonferroni correction was applied. To compare classification performance between *Labyrinth Oddball* scenario and *Armrest* scenario, the data were analyzed in two steps. First, the mean of classification performance was calculated for each subject. Such subject's means were calculated separately for each of the scenarios, which were used for the dependent variables for the statistical test.

Second, two different scenarios were compared by using the Mann-Whitney U test: 1) The median of the subject's means was calculated for each scenario and 2) The mean rank for each scenario was calculated. Mann-Whitney U test was performed on subject's means for each scenario to compare two different scenarios. Note that different subjects participated in both experiments (*Labyrinth Oddball*/*Armrest*) except for one subject (coded as subject 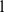 for the *Labyrinth Oddball* and subject 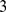 for the *Armrest* scenario).

#### ML Analysis: Detection of Movement Intention

Again, all ML evaluations have been performed using the open source signal processing and classification environment pySPACE [66]. To detect movement intention BR classifies two classes: (i) *no movement preparation* and (ii) *movement preparation*. For correct labeling of both classes during training and for performance evaluation the problem of *ambiguous* instances emerges here, i.e., windows that neither clearly belong to the *movement preparation* nor to the *no movement preparation* class. To deal with this problem, training is performed time dependent on the lock-out event, i.e., only specific windows were used for training. Since movement intention is neither locked to a certain stimulus (i.e., command) nor happens after a fixed time of delay, it is necessary to *continuously* analyze the EEG stream during test. This continuous analysis is based on a sliding window approach, i.e., a window of a fixed length is extracted every 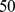 ms from the EEG stream.

For offline evaluation, an approach similar to the one presented in [54] was chosen: Windows for the *movement preparation* class had a length of 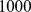 ms and were cut out with respect to the movement marker. For training, 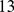 different windows were analyzed for that class that ended between 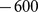 to 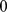 ms relative to the movement marker, i.e., [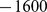, 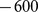], [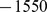, 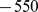], [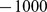, 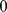]. Training windows for the *no movement preparation* class were cut out every 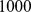 ms, if no other marker was stored in the data stream 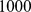 ms before or 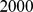 ms after that window. Since the duration of a rest period was not fixed, the number of instances per data set differed for that class (from 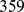 to 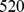). For testing, sliding windows were cut out every 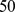 ms in the range from 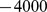 to 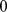 ms. Data processing in both cases (training and test) was done as follows: All trials were standardized (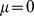, 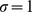), decimated to 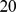 Hz and band pass filtered (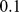
–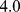 Hz). Only the last 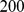 ms were used for feature generation: 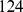 channels 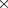 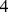 time points 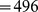 features. Finally, a SVM was trained on the feature vectors of the training data. In each training run, SVM parameters were optimized with an internal 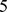-fold cross validation using a pattern search algorithm [69].

**Figure 13:**
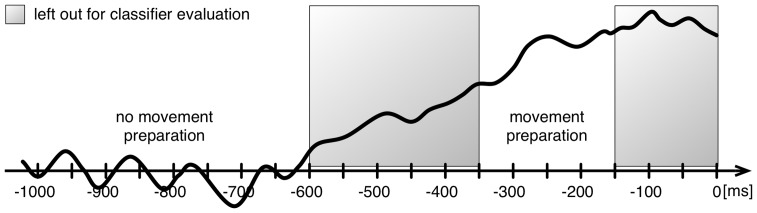
Classifier evaluation for sliding windows. It is illustrated how evaluation was performed in the sliding window approach. Evaluation depends on the end time of a sliding window: (i) less than 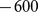 ms: true label “no movement preparation”, (ii) between 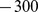 to 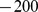 ms: true label “movement preparation”, (iii) in gray shaded area: left out for evaluation due to unknown true label or already started movement. The black line illustrates the average ERP difference curve for channels C3/C4 over all subjects.

For classifier evaluation, a 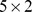-fold cross validation was used for each subject on the merged data of one session (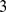 concatenated sets). To calculate a performance measure (BA), labeling of the sliding windows was required. Since the onset of the LRP cannot exactly be determined for single trials (see explanations given above), we defined a time range from 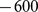 to 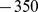 ms based on average ERP analysis (see Fig. 13) as an uncertain area, i.e., as a time range in which we could not be certain (for each single trial) whether or not the brain was already preparing a movement. Sliding windows ending in this time interval were left out for performance calculation. Also, predictions based on windows ending at 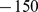 to 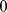 ms (see Fig. 13) were excluded due to the fact that the actual movement onset happens before the movement marker is stored (see estimation of movement onset in Sec. “sec:ArmrestResultsBehavior”).

Since the training windows overlapped in time, similar performances for consecutive windows were expected. Hence, overlapping windows were analyzed for each subject in order to find points in time which lead to significantly different performances to define borders of clusters. To evaluate *which* combination of two training windows is optimal, performance of all possible combinations of two training windows were computed, i.e., combinations within the same cluster and between clusters. The mean performance of all within – cluster combinations and all between-cluster combinations for each defined cluster, the mean performances of the single training windows in each cluster, and the performance of all training windows were finally compared using repeated measures ANOVA with *combination* (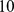 levels) as a within-subjects factor. Here, performance for all 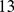 training windows served as a baseline, representing the case that no specific training windows were chosen.

### Results

#### Behavior

In the whole experiment, subjects responded in total to 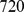 target stimuli and missed 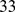 target stimuli (mean and standard deviation across subjects for omission errors: 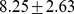). This low amount of omission errors, i.e., *missed targets*, was expected due to the low effort of the main task. In total 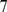 commission errors on standard stimuli occurred (subject 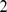: 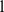 commission error, subject 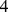: 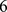 commission errors). The response time was on average 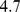 sec (mean of subject's median), with a median minimal response time of 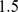 sec and a median maximal response time of 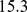 sec. The difference between minimal and maximal response time was between 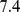 sec and 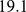 sec (median 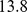 sec). A rest period of at least 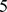 sec preceded on average 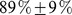 of the performed movements. For EMG onset detection only the data from M. biceps brachii contained usable information. However, we observed a preload in muscle activity in the EMG recordings of one subject resulting in an EMG onset detection at around 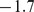 sec relative to the movement marker. Therefore, EMG onset was not used to determine movement onset. Instead, movement onset was estimated based on the analysis of motion tracking data recorded during intentional movements of the right arm in a study performed in [62]. We calculated the time it took to move the arm by 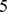 cm from the rest position. For the subjects recorded in [62] such movement took on average 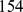 ms. Based on this analysis, we assumed that the physical movement onset in this very similar setup was around 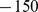 ms relative to the movement marker.

#### Performance of BR in the Detection of a Highly Underrepresented State

**Figure 14:**
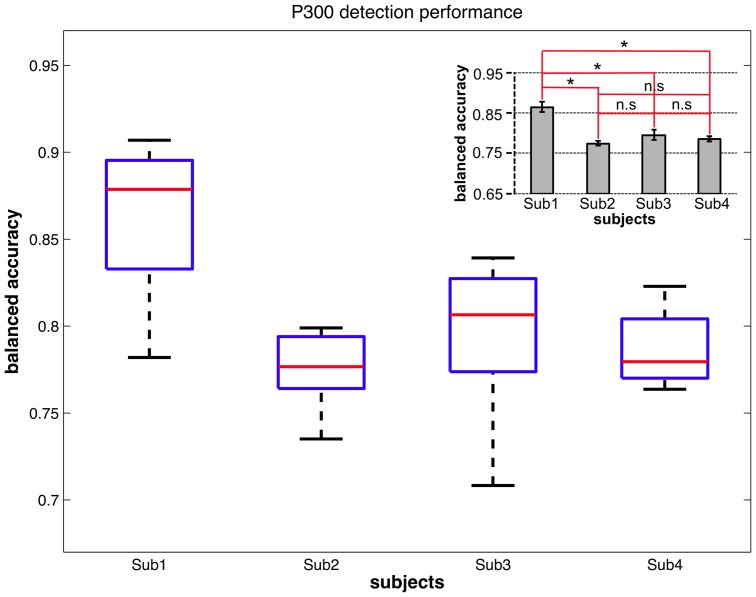
Classification performance in the Armrest scenario. Results for the performance of the classifier trained in the dual BR scenario for the classification of *missed target* vs. *target* instances after classifier transfer are shown for all subjects individually. The red lines in the main diagram mark the median values of obtained classification performance for each subject. The inserted diagram illustrates mean classification performance values and standard error of mean (SEM). Highest classification performance is observed for subject 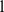.

The resulting BA values are shown in Fig. 14. Best classification performance was obtained for subject 1. Mean classification performance was slightly lower compared to the *Labyrinth Oddball* scenario (see Fig. 14 vs. Fig. 11). However, classification performances between both scenarios did not differ significantly [median for *Labyrinth Oddball*: 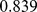, median for *Armrest*: 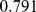, mean ranks of *Labyrinth Oddball*: 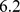, mean ranks of *Labyrinth Oddball*: 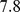, 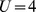, 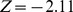, 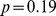, 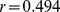]. These results show that our classifier transfer approach can be applied to realistic scenarios in which the subject is performing several tasks but is not always allowed to respond to an important stimulus straight away. Moreover, we were successful in classifying a highly underrepresented class.

#### Combination of Training Windows for a Robust Detection of Movement Intention

**Figure 15:**
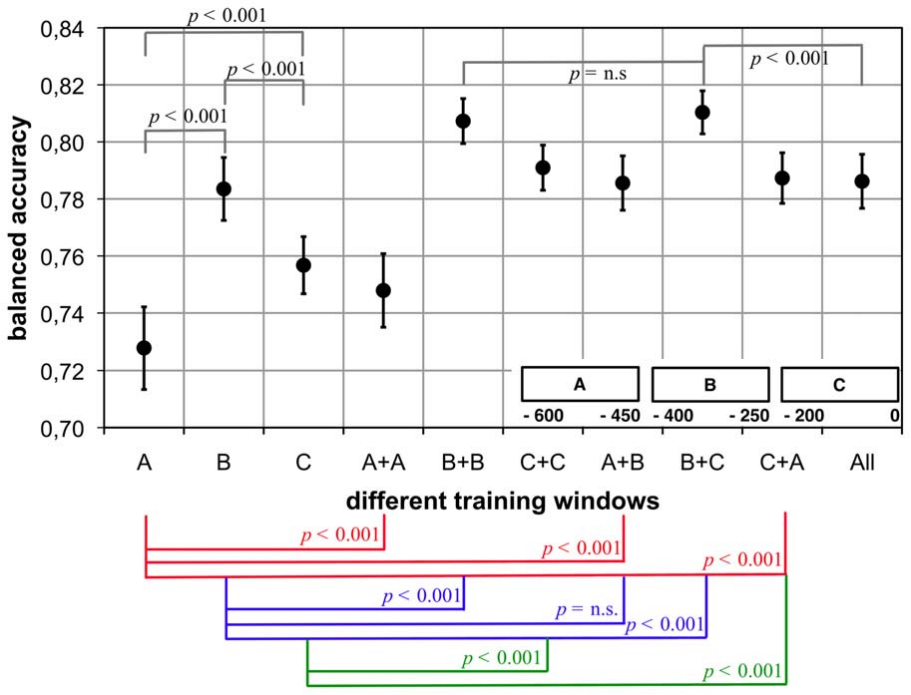
Method illustration and performance for different training windows. The diagram illustrates the combination of training time of two windows using the previously found clusters (see methods description for details), classification performance and statistics. Classification performance of a 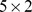-fold cross validation for four subjects quantified with mean BA and standard error is presented by the dots in the diagram. The x-axis shows different training settings: A, B, C – one training window per movement marker ending at different times with respect to the movement marker; A+A, B+B, C+C, A+B, B+C, C+A – two training windows per movement marker, combined within the same cluster or with other clusters. All – all 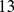 training windows were used to train a classifier.

Based on the statistical analysis that was performed to find time points which lead to significantly different performances to define borders of clusters, 13 training windows were grouped in three *clusters*: early [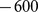, 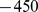] ms, middle [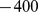, 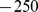] ms and late [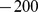, 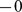] ms. Across all subjects, the middle cluster (cluster B) provided a significantly better classification performance compared to both the early (cluster A) and late cluster (cluster C): B vs. A: 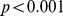, B vs. C: 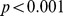 (see also first three columns of Fig. 15). Further, classification performance was significantly higher when using training windows of the late cluster than when using training windows of the early cluster (C vs. A: 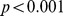). Figure 15 depicts a comparison of classifiers trained on one, two or else all 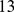 training windows.

Results showed that the combination of two training windows increased classification performance (A+A vs. A: 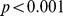, B+B vs. B: 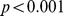, C+C vs. C: 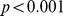, A+B vs. A: 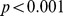, B+C vs. B: 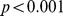, B+C vs. C: 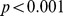, C+A vs. A: 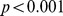, C+A vs. C: 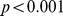) except when combining training windows from B and A in comparison to the performance when using single windows from B (A+B vs. B: 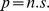). The overall best performance was obtained when combining training windows from cluster B and C, although there was no significant difference compared to window combinations within B (B+C vs. B+B: 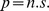). The average TPR from training window combination of cluster B and C at time point 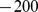 ms (latest time point of movement preparation class and before estimated movement onset at 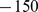 ms, see Fig. 13 and Sec. “sec:ArmrestResultsBehavior”) was on average 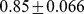. For the application in the exoskeleton this would mean a correct modulation of the control in 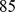 out of 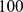 movements. Performance of a classifier trained on all 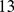 training windows was worse than that of the classifier trained on the best pair of windows (All vs. B+C: 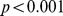).

### Discussion: Dual BR Scenario Armrest

In the experiments performed in the *Armrest* scenario we showed that our approach of classifier transfer for the detection of the recognition of important information, which was developed in the *Labyrinth Oddball* scenario, can be transferred into a new setup in which two tasks had to be performed that influence each other while still achieving similar classification performance. We confirmed our results from Part. “sec:BrioOddball” that substitution of training examples and hence, partly classifier transfer between two different classes in training and test is possible. Our results indicate that the supervision of trained operators in a teleoperation scenario is feasible, since *trained* operators will miss only few examples of target stimuli similar to subjects that are performing a simple task in the *Armrest* scenario (compared to the more demanding *Labyrinth Oddball* scenario). In both cases only very few examples for missed target stimuli were available. These few examples could not have been sufficient for alternative training methods that allow direct training with few training examples as already shown for the *Labyrinth Oddball* setup (see [74]).

Besides the detection of the success of information recognition we showed that the detection of movement intention based on EEG data in the ERP range [75]–[78] is possible under dual task conditions in which the subjects are not solely concentrating on movement preparation. Within the sliding window analysis we investigated the influence of different training windows for classifier construction on the performance when continuously predicting upcoming movements. We found subject-independent time intervals, which provide different detection rates depending on which interval the training window belongs to. This allows more general suggestions for classifier training on data of new subjects, like using a first training window ending between 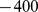 to 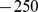 ms before movement marker (cluster B) and a second one between 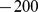 to 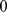 ms (cluster C). For our experiments we estimated the actual movement onset at around 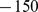 ms before the movement marker. However, this is only a rough approximation since the movement marker was set after 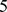 cm movement in one direction. Still, results of time intervals with significantly different performance remain and are valid.

The choice of the training windows critically depends on the point in time when the movement has to be predicted (e.g., in a range from 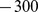 to 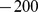 ms relative to the movement marker (Fig. 13)). If the application requires an earlier prediction, this may have an influence on the choice of the optimal training window. Exact interval boundaries for choosing appropriate training windows for movement prediction remain to some degree subject-specific. However, based on the results obtained here, large subject-specific differences are not expected.

To summarize, with the experiments conducted in the dual BR scenario *Armrest*, we showed that: (i) the intentional state “movement intention” as well as (ii) the cognitive state “recognition of important stimuli and task coordination” can be detected in single trial by BR while a subject is performing a dual-task that is similar to the described dual-task that has to be performed during the teleoperation of a robot. The classification of the different states resulted in high performance. Reliability of the detection of movement preparation processes could be improved by combining appropriate training windows. Classification of missed target instances versus target instances was made possible by applying our approach of classifier transfer. The reliable and high performance in single trial prediction of the dual BR we obtained is an important prerequisite for our approach of adapting both the OMS and exoskeleton control with respect to the changing requirements of the user.

## Conclusions

The recording, analysis, and integration of (psycho-)physiological data to adapt human-machine interfaces with respect to changing intentional or cognitive states and behavior of the user is a promising way to improve the functionality of technical systems that are interacting with the user [30]. We presented two different scenarios here to investigate the application of BR. In the *Labyrinth Oddball* scenario we showed that BR, i.e., the detection of human states (here the cognitive state of “recognition of important stimuli and task coordination”) based on ML analysis of the EEG, is possible. This result is supported by the finding that results of average P300 analysis is predictive for performance of ML analysis for P300 detection and vice versa. In the *Armrest* scenario two different states (“movement intention” and “recognition of important stimuli and task coordination”) could successfully and simultaneously be detected by a *dual* BR approach while a human was interacting in this scenario, which was very similar to the teleoperation scenario.

In the *Labyrinth Oddball* scenario we developed an approach for classifier transfer in which a training class with few examples (missed targets) is substituted by a training class with many examples (standards). Instances of both classes could be shown to evoke very similar but not the same ERP activity. Here the missing of a pronounced ERP activity, namely the P300, was sufficient to allow the transfer. Hence, we showed that classifier transfer is not only possible between classes of samples that contain the same or very similar ERPs evoked by the same or at least very similar brain processes (see [60], [61]). More important for the success of classifier transfer in our example is the absence of some brain process, i.e., target recognition processes, than the similarity of brain processes. Note that the absence of these processes can have a number of different causes that were not further investigated here. Only for subjects with clear absence of P300 on missed targets compared to targets and very similar shape of the average ERP forms on missed targets and standards, a transfer of a classifier (trained on standard stimuli to later detect missed targets) resulted in high classification performance. The classifier transfer could also be applied successfully in the *Armrest setup* which produces only very few examples for the infrequent but important class (missed targets) as it is expected to be the case for trained operators in teleoperation scenarios. Small amounts of training data are not sufficient for direct training, but classifier transfer allows to apply BR in such an application-oriented scenario. Furthermore, we showed for both applications of BR that classification performance can be improved significantly independent of the subject by combining training windows identified to likely contain important information for classification.

To conclude, our work shows that BR can be applied during complex human-machine interaction, since brain patterns that are detected by single trial ML analysis can be correlated to specific activities of the brain, as shown for ERP activity, and hence correlated with specific states of the operator. The gained knowledge about the occurrence of such states can then be used to infer upcoming behavior by means of eBR [30]. The knowledge gained about upcoming behavior is a basic requirement for the implementation of predictive HMIs that better support upcoming interaction and thus improve human-machine interaction as explained by the example of robotic teleoperation. Earlier investigations with simulated adaptation of an exoskeleton control by eBR showed that our concept of adapting the control of the exoskeleton for robot teleoperation does indeed help to reduce the effort of the user during interaction [52]. Further, results of a recently conducted online study in the teleoperation scenario [49], [53] showed that our approach can successfully be applied *online* and be fully integrated for the adaptation of the exoskeleton and the OMS as proposed in this paper. To implement predictive HMIs, BR has to be embedded into an application as formally described in [30]. For this it is not only required to automatically describe interaction rules and behavior of the interacting human as discussed in [30] but to particularly understand the nature of detected brain patterns during complex interaction. That the later is possible was shown in the work presented here.

## Supporting Information

Video S1 **Online adaptation of the OMS by eBR in the teleoperation scenario.** It is shown how the OMS that is adapted by eBR supports the current state, i.e., success or failure in the recognition of important information, of an operator who is teleoperating a robotic arm. In case a failure in the recognition of important, i.e., task-relevant, information is detected, the important information is repeated after a short while. In case that success in the recognition of important information was detected, the important information will not be repeated for a longer time during which a response of the subject is monitored. In case the response is missing within the extended response time the important information is repeated although BR detected success in the recognition of the important, i.e., task-relevant, information.
(MP4)

Video S2 **Teleoperation scenario and eBR for the adaptation of two HMIs.** It is shown how an operator controls a robotic arm via a virtual scenario that is presented to him by an HMI. The control of the robotic arm is enabled by an exoskeleton. While controlling the robotic arm, the operator has to respond to important warnings. The implemented OMS is supporting the operator in this task. Both HMIs, the OMS and the exoskeleton, are adapted by eBR.
(MP4)

Video S3 **Online adaptation of the exoskeleton by eBR in the teleoperation scenario.** It is shown how the exoskeleton's control is adapted by eBR to ease the lock out from a rest position. Online prediction values and the point in time at which sensors that are integrated in the exoskeleton detect the movement onset are visualized in an inserted diagram. Video and online prediction values for BR as well as movement onsets are synchronized in time. The video shows that too early or false movement predictions by BR are irrelevant for the control of the system. Only correct movement predictions ease the handling of the exoskeleton by the operator.
(MP4)

Video S4 **Online detection of failure and success in the recognition of important information in the Labyrinth oddball scenario.** It is shown how BR is able to detect the success and failure in the recognition of important, i.e., task-relevant, information. P300 related processes that are evoked by target recognition processes are detected online in the Labyrinth Oddball scenario.
(MP4)

## References

[1] Young JE, Hawkins R, Sharlin E, Igarashi T (2009) Toward acceptable domestic robots: Applying insights from social psychology. Int J Soc Robot 1: 95–108.

[2] Kaupp T, Makarenko A, Durrant-Whyte H (2010) Human-robot communication for collaborative decision making making – A probabilistic approach. Rob Auton Syst 58: 444–456.

[3] Prodanov P, Drygajlo A (2005) Bayesian networks based multi-modality fusion for error handling in human-robot dialogues under noisy conditions. Speech Communication 45: 231–248.

[4] Graf B, Parlitz C, Hägele M (2009) Robotic home assistant Care-O-bot ® product vision and innovation platform. In: Proceedings of the 13th International Conference on Human-Computer Interaction. Part II: Novel Interaction Methods and Techniques. Berlin, Heidelberg: Springer-Verlag, 312–320. doi:10.1007/978-3-642-02577-8 34

[5] Kurihara K, Sugiyama D, Matsumoto S, Nishiuchi N, Masuda K (2009) Facial emotion and gesture reproduction method for substitute robot of remote person. Computers and Industrial Engineering 56: 631–647.

[6] Allanson J, Fairclough S (2004) A research agenda for physiological computing. Interact Comput 16: 857–878.

[7] Woods DD (1996) Decomposing Automation: Apparent Simplicity, Real Complexity, CRC, chapter. 1: 3–17.

[8] Parra L, Spence C, Gerson A, Sajda P (2003) Response error correction-a demonstration of improved human-machine performance using real-time EEG monitoring. IEEE Trans Neural Syst Rehabil Eng 11: 173–177. 12899266 10.1109/TNSRE.2003.814446

[9] Zander TO, Gaertner M, Kothe C, Vilimek R (2010) Combining Eye Gaze Input With a Brain-Computer Interface for Touchless Human-Computer Interaction. Int J Hum Comput Interact 27: 38–51.

[10] Wolpaw JR, Birbaumer N, McFarland DJ, Pfurtscheller G, Vaughan TM (2002) Brain-computer interfaces for communication and control. Clin Neurophysiol 113: 767–791. 12048038 10.1016/s1388-2457(02)00057-3

[11] Guger C, Harkam W, Hertnaes C, Pfurtscheller G (1999) Prosthetic control by an EEG-based brain-computer interface (BCI). In: Proceedings of the 5th European AAATE Conference. 3–6.

[12] Birbaumer N (2006) Breaking the silence: brain-computer interfaces (BCI) for communication and motor control. Psychophysiology 6: 517–532. 10.1111/j.1469-8986.2006.00456.x 17076808

[13] Leeb R, Keinrath C, Friedman D, Guger C, Scherer R, et al (2006) Walking by thinking: the brainwaves are crucial, not the muscles! Presence: Teleoperators and Virtual Environments. 15: 500–514.

[14] Enzinger C, Ropele S, Fazekas F, Loitfelder M, Gorani F, et al (2008) Brain motor system function in a patient with complete spinal cord injury following extensive brain-computer interface training. Exp Brain Res 190: 215–223. 18592230 10.1007/s00221-008-1465-y

[15] Blankertz B, Dornhege G, Krauledat M, Müller K, Kunzmann V, et al (2006) The Berlin Brain-Computer Interface: EEG-based communication without subject training. IEEE Trans Neural Syst Rehabil Eng 14: 147–152. 16792281 10.1109/TNSRE.2006.875557

[16] Farwell LA, Donchin E (1988) Talking off the top of your head: toward a mental prosthesis utilizing event-related brain potentials. Electroencephalogr Clin Neurophysiol 70: 510–523. 2461285 10.1016/0013-4694(88)90149-6

[17] Blankertz B, Dornhege G, Krauledat M, Schröder M, Williamson J, et al.. (2006) The Berlin Brain-Computer Interface presents the novel mental typewriter Hex-o-Spell. In: Verlag der Technischen Universität Graz. 108–109.

[18] Allison B, Gräser A, Graimann B (2007) Why use a BCI if you are healthy? In: ACE Workshop-Brain Computer Interfaces and Games. 7–11.

[19] Reuderink B (2008) Games and Brain-Computer Interfaces: The State of the Art. WP2 BrainGain Deliverable HMI University of Twente September 2008: 1–11.

[20] Nijholt A, Tan D, Allison BZ, Millán JdR, Graimann B (2008). Brain-computer interfaces for HCI and games.

[21] Summerer L, Izzo D, Rossini L, editors (2009) Brain Machine Interfaces for Space Applications: Enhancing Astronaut Capabilities, volume 86 of International Review of Neurobiology. New York: Academic Press.

[22] Millán JdR, Galán F, Vanhooydonck D, Lew E, Philips J, et al.. (2009) Asynchronous non-invasive brain-actuated control of an intelligent wheelchair. In: Proc. 31st Annu. Conf. IEEE Eng. Med. Biol. Soc. Minneapolis, MN, 3361–3364. 10.1109/IEMBS.2009.5332828 19963794

[23] Trieu HT, Willey K, Nguyen HT (2009) Adaptive shared control strategies based in the Bayesian recursive technique in an intelligent wheelchair. In: Proc. 31st Annu. Conf. IEEE Eng. Med. Biol. Soc. Minneapolis, MN, 7118–7121. 10.1109/IEMBS.2009.5332876 19963949

[24] Cutrell E, Tan D (2008) Bci for passive input in hci. In: CHI 2008– Workshop Brain-Computer Interfaces for HCI and Games. 1–3.

[25] Zander TO, Kothe C, Jatzev S, Gärtner M (2010) Enhancing human-computer interaction with input from active and passive brain-computer interfaces. Brain-Computer Interfaces: 181–199.

[26] George L, Lécuyer A (2010) An overview of research on “passive” brain-computer interfaces for implicit human-computer interaction. In: International Conference on Applied Bionics and Biomechanics ICABB 2010– Workshop W1 “Brain-Computer Interfacing and Virtual Reality”. Venice, Italy.

[27] Haufe S, Treder MS, Gugler MF, Sagebaum M, Curio G, et al (2011) EEG potentials predict upcoming emergency brakings during simulated driving. J Neural Eng 8: 056001. 21799241 10.1088/1741-2560/8/5/056001

[28] Pfurtscheller G, Allison B, Brunner C, Bauernfeind G, Solis-Escalante T, et al (2010) The hybrid BCI. Front Neurosci 4: 1–11. 20582271 10.3389/fnpro.2010.00003 PMC2891647

[29] Allison BZ, Leeb R, Brunner C, Müller-Putz GR, Bauernfeind G, et al (2012) Toward smarter BCIs: extending BCIs through hybridization and intelligent control. J Neural Eng 9: 013001. 22156029 10.1088/1741-2560/9/1/013001

[30] Kirchner EA, Drechsler R (2013) A Formal Model for Embedded Brain Reading. Industrial Robot: An International Journal 40: 530–540.

[31] Coles M (1989) Modern mind-brain reading: Psychophysiology, physiology, and cognition. Psychophysiology 26: 251–269. 2667018 10.1111/j.1469-8986.1989.tb01916.x

[32] Haynes JD, Rees G (2006) Decoding mental states from brain activity in humans. Nat Rev Neurosci 7: 523–534. 16791142 10.1038/nrn1931

[33] Kamitani Y, Tong F (2005) Decoding the visual and subjective contents of the human brain. Nature Neuroscience 8: 679–685. 15852014 10.1038/nn1444 PMC1808230

[34] Miyawaki Y, Uchida H, Yamashita O, Sato Ma, Morito Y, et al (2008) Visual Image Reconstruction from Human Brain Activity using a Combination of Multiscale Local Image Decoders. Neuron 60: 915–929. 19081384 10.1016/j.neuron.2008.11.004

[35] Naselaris T, Prenger RJ, Kay KN, Oliver M, Gallant JL (2009) Bayesian Reconstruction of Natural Images from Human Brain Activity. Neuron 63: 902–915. 19778517 10.1016/j.neuron.2009.09.006 PMC5553889

[36] Polyn SM, Natu VS, Cohen JD, Norman KA (2005) Category-specific cortical activity precedes retrieval during memory search. Science (New York, NY) 310: 1963–1966. 10.1126/science.1117645 16373577

[37] Suppes P, Perreau-Guimaraes M, Wong DK (2009) Partial orders of similarity differences invariant between EEG-recorded brain and perceptual representations of language. Neural Comput 21: 3228–3269. 19686069 10.1162/neco.2009.04-08-764

[38] Isreal J, Chesney G, Wickens C, Donchin E (1980) P300 and tracking diffculty: Evidence for multiple resources in dual-task performance. Psychophysiology 17: 259–273. 7384376 10.1111/j.1469-8986.1980.tb00146.x

[39] Squires NK, Squires KC, Hillyard SA (1975) Two varieties of long-latency positive waves evoked by unpredictable auditory stimuli in man. Electroencephalogr Clin Neurophysiol 38: 387–401. 46819 10.1016/0013-4694(75)90263-1

[40] Verleger R, Heide W, Butt C, Kömpf D (1994) Reduction of P3b in patients with temporo-parietal lesions. Brain Res Cogn Brain Res 2: 103–116. 7833690 10.1016/0926-6410(94)90007-8

[41] Polich J (2007) Updating P300: an integrative theory of P3a and P3b. Clin Neurophysiol 118: 2128–2148. 17573239 10.1016/j.clinph.2007.04.019 PMC2715154

[42] Kutas M, McCarthy G, Donchin E (1977) Augmenting mental chronometry: the P300 as a measure of stimulus evaluation time. Science 197: 792–795. 887923 10.1126/science.887923

[43] Salisbury D, Rutherford B, Shenton M, McCarley R (2001) Button-pressing Affects P300 Amplitude and Scalp Topography. Clin Neurophysiol 112: 1676–1684. 11514251 10.1016/s1388-2457(01)00607-1 PMC2650488

[44] Kirchner EA, Metzen JH, Duchrow T, Kim SK, Kirchner F (2009) Assisting Telemanipulation Operators via Real-Time Brain Reading. In: Lohweg V, Niggemann O, editors, Proc. Mach. Learning in Real-time Applicat. Workshop 2009. Paderborn, Germany, Lemgoer Schriftenreihe zur industriellen Informationstechnik.

[45] Rolke B, Heil M, Streb J, Hennighausen E (2001) Missed prime words within the attentional blink evoke an n400 semantic priming effect. Psychophysiology 38: 165–174. 11347861

[46] West R (2011) The temporal dynamics of prospective memory: a review of the ERP and prospective memory literature. Neuropsychologia 49: 2233–2245. 21187107 10.1016/j.neuropsychologia.2010.12.028

[47] Kirchner EA, Kim SK (2012) EEG in Dual-Task Human-Machine Interaction: Target Recognition and Prospective Memory. In: Proceedings of the 18th Annual Meeting of the Organization for Human Brain Mapping.

[48] Kirchner EA, Kim SK, Fahle M (2013) EEG in Dual-Task Human-Machine Interaction: On the Feasibility of EEG based Support of Complex Human-Machine Interaction. Perception 42 ECVP Abstract Supplement: 220.

[49] Woehrle H, Kirchner EA (2014) Online Detection of P300 related Target Recognition Processes During a Demanding Teleoperation Task. In: Proc. International Conference on Physiological Computing Systems, (PhyCS 2014). Lissabon, Portugal: SCITEPRESS Digital Library.

[50] Folgheraiter M, Bongardt B, Schmidt S, de Gea Fernandéz J, Albiez J, et al.. (2009) Design of an Arm Exoskeleton using an hybrid Motion-Capture and Model-Based Technique. In: IEEE Int. Conf. Robotics and Automation. Kobe.

[51] Folgheraiter M, Bongardt B, Albiez J, Kirchner F (2008) A bio-inspired haptic interface for telerobotics applications. In: IEEE Int. Conf. Robotics and Biomemetics. Bangkok, 560–565.

[52] Folgheraiter M, Jordan M, Straube S, Seeland A, Kim S, et al (2012) Measuring the improvement of the interaction comfort of a wearable exoskeleton. International Journal of Social Robotics 4: 285–302.

[53] Seeland A, Wöhrle H, Straube S, Kirchner EA (2013) Online movement prediction in a robotic application scenario. In: 6th International IEEE EMBS Conference on Neural Engineering (NER). San Diego, California, 41–44.

[54] Kirchner EA, Albiez JC, Seeland A, Jordan M, Kirchner F (2013) Towards assistive robotics for home rehabilitation. In: Proceedings of the International Conference on Biomedical Electronics and Devices. SciTePress, 168–177.

[55] Kornhuber HH, Deecke L (1965) Hirnpotentialänderungen bei Willkürbewegungen und passiven Bewegungen des Menschen: Bereitschaftspotential und reafferente Potentiale. Pflügers Archives 284: 1–17. 14341490

[56] Deecke L, Scheid P, Kornhuber HH (1969) Distribution of readiness potential, pre-motion positivity, and motor potential of the human cerebral cortex preceding voluntary finger movements. Exp Brain Res 7: 158–568. 5799432 10.1007/BF00235441

[57] Balconi E (2009) The multicomponential nature of movement-related cortical potentials: functional generators and psychological factors. Neurophysiol Trends 5: 59–84.

[58] Miller N (1969) Learning of visceral and gladular responses. Science 163: 434–445. 5812527 10.1126/science.163.3866.434

[59] Pan SJ, Yang Q (2010) A Survey on Transfer Learning. IEEE Trans Knowl Data Eng 22: 1345–1359.

[60] Iturrate I, Montesano L, Minguez J (2013) Task-dependent signal variations in EEG error-related potentials for brain-computer interfaces. J Neural Eng 10: 026024. 23528750 10.1088/1741-2560/10/2/026024

[61] Kim SK, Kirchner EA (2013) Classifier transferability in the detection of error related potentials from observation to interaction. In: Proceedings of the IEEE International Conference on Systems, Man, and Cybernetics, SMC 2013, Manchester, UK, October 13–16, 2013.

[62] Tabie M, Kirchner EA (2013) EMG onset detection – Comparison of different methods for a movement prediction task based on emg. In: Alvarez S, Solé-Casals J, Fred A, Gamboa H, editors, Proceedings of the 6th International Conference on Bio-inspired Systems and Signal Processing (BIOSIGNALS-13). Barcelona: SciTePress, 242–247.

[63] Kaufmann T, Hammer EM, Kübler A (2011) ERPs contributing to classification in the P300 BCI. In: 5th International BCI Conference 2011: 136–139.

[64] Jansen B, Allam A, Kota P, Lachance K, Osho A, et al (2004) An exploratory study of factors affecting single trial P300 detection. IEEE Trans Biomed Eng 51: 975–978. 15188867 10.1109/TBME.2004.826684

[65] Ghaderi F, Kim SK, Kirchner EA (2014) Effects of eye artifact removal methods on single trial P300 detection, a comparative study. Journal of Neuroscience Methods 221: 41–47. 24056231 10.1016/j.jneumeth.2013.08.025

[66] Krell MM, Straube S, Seeland A, Wöhrle H, Teiwes J, et al. (2013). pySPACE. Available: https://github.com/pyspace. 10.3389/fninf.2013.00040 PMC3871959 24399965

[67] Chang CC, Lin CJ (2001) LIBSVM: a library for support vector machines. Available: http://www.csie.ntu.edu.tw/cjlin/libsvm.

[68] Hanley JA, McNeil BJ (1982) The meaning and use of the area under a receiver operating characteristic (ROC) curve. Radiology 143: 29–36. 7063747 10.1148/radiology.143.1.7063747

[69] Nocedal J, Wright S (1999) Numerical Optimization. Springer Series in Operations Research. Springer.

[70] Brodersen K, Ong C, Stephan K, Buhmann JM (2010) The balanced accuracy and its posterior distribution. Proceedings of the 20th International Conference on Pattern Recognition: 3121–3124.

[71] Krauledat M, Tangermann M, Blankertz B, Müller K (2008) Towards zero training for Brain-Computer interfacing. PLoS ONE 3: e2967. 18698427 10.1371/journal.pone.0002967 PMC2500157

[72] Fazli S, Popescu F, Danóczy M, Blankertz B, Müller K, et al (2009) Subject-independent mental state classification in single trials. Neural Netw 22: 1305–1312. 19560898 10.1016/j.neunet.2009.06.003

[73] Lotte F, Guan C (2010) Learning from other subjects helps reducing Brain-Computer interface calibration time. In: ICASSP. 614–617.

[74] Metzen JH, Kim SK, Kirchner EA (2011) Minimizing calibration time for brain reading. In: Mester R, Felsberg M, editors, Pattern Recognition. Springer Berlin/Heidelberg, volume 6835 of Lecture Notes in Computer Science, 366–375.

[75] Blankertz B, Dornhege G, Lemm S, Krauledat M, Curio G, et al (2006) The Berlin Brain-Computer Interface: Machine learning based detection of user specific brain states. J Univers Comput Sci 12: 581–607.

[76] Bai O, Rathi V, Lin P, Huang D, Battapady H, et al (2011) Prediction of human voluntary movement before it occurs. Clin Neurophysiol 122: 364–372. 20675187 10.1016/j.clinph.2010.07.010 PMC5558611

[77] Ibez J, Serrano J, del Castillo M, Barrios L, Gallego J, et al.. (2011) An EEG-Based Design for the Online Detection of Movement Intention. Adv Neural Inf Process Syst: 370–377.

[78] Wang B, Wan F (2009) Classification of Single-Trial EEG based on support vector clustering during finger movement. Advances in Neural Networks-ISNN 2009: 354–363.

